# Functional and Bioinformatics Analysis of Two *Campylobacter jejuni* Homologs of the Thiol-Disulfide Oxidoreductase, DsbA

**DOI:** 10.1371/journal.pone.0106247

**Published:** 2014-09-02

**Authors:** Anna D. Grabowska, Ewa Wywiał, Stanislaw Dunin-Horkawicz, Anna M. Łasica, Marc M. S. M. Wösten, Anna Nagy-Staroń, Renata Godlewska, Katarzyna Bocian-Ostrzycka, Katarzyna Pieńkowska, Paweł Łaniewski, Janusz M. Bujnicki, Jos P. M. van Putten, E. Katarzyna Jagusztyn-Krynicka

**Affiliations:** 1 Department of Bacterial Genetics, Institute of Microbiology, University of Warsaw, Warsaw, Poland; 2 Laboratory of Bioinformatics and Protein Engineering, International Institute of Molecular and Cell Biology, Warsaw, Poland; 3 Department of Infectious Diseases and Immunology, Utrecht University, Utrecht, the Netherlands; 4 World Health Organization Collaborating Centre for Reference and Research on Campylobacter/ World Organisation for Animal Health Reference Laboratory for Campylobacteriosis, Utrecht, The Netherlands; 5 Institute of Science and Technology, Klosterneuburg, Austria; 6 Institute of Molecular Biology and Biotechnology, Faculty of Biology, Adam Mickiewicz University, Poznan, Poland; Instituto de Biociencias - Universidade de São Paulo, Brazil

## Abstract

**Background:**

Bacterial Dsb enzymes are involved in the oxidative folding of many proteins, through the formation of disulfide bonds between their cysteine residues. The Dsb protein network has been well characterized in cells of the model microorganism *Escherichia coli*. To gain insight into the functioning of the Dsb system in epsilon-Proteobacteria, where it plays an important role in the colonization process, we studied two homologs of the main *Escherichia coli* Dsb oxidase (EcDsbA) that are present in the cells of the enteric pathogen *Campylobacter jejuni*, the most frequently reported bacterial cause of human enteritis in the world.

**Methods and Results:**

Phylogenetic analysis suggests the horizontal transfer of the epsilon-Proteobacterial DsbAs from a common ancestor to gamma-Proteobacteria, which then gave rise to the DsbL lineage. Phenotype and enzymatic assays suggest that the two *C. jejuni* DsbAs play different roles in bacterial cells and have divergent substrate spectra. CjDsbA1 is essential for the motility and autoagglutination phenotypes, while CjDsbA2 has no impact on those processes. CjDsbA1 plays a critical role in the oxidative folding that ensures the activity of alkaline phosphatase CjPhoX, whereas CjDsbA2 is crucial for the activity of arylsulfotransferase CjAstA, encoded within the *dsbA2-dsbB-astA* operon.

**Conclusions:**

Our results show that CjDsbA1 is the primary thiol-oxidoreductase affecting life processes associated with bacterial spread and host colonization, as well as ensuring the oxidative folding of particular protein substrates. In contrast, CjDsbA2 activity does not affect the same processes and so far its oxidative folding activity has been demonstrated for one substrate, arylsulfotransferase CjAstA. The results suggest the cooperation between CjDsbA2 and CjDsbB. In the case of the CjDsbA1, this cooperation is not exclusive and there is probably another protein to be identified in *C. jejuni* cells that acts to re-oxidize CjDsbA1. Altogether the data presented here constitute the considerable insight to the Epsilonproteobacterial Dsb systems, which have been poorly understood so far.

## Introduction

Bacterial proteins of the Dsb (disulfide bond) system catalyze the formation of disulfide bridges, a post-translational modification of extra-cytoplasmic (periplasm-located, membrane-anchored or secreted) proteins, which leads to stabilization of their tertiary and quaternary structures and often influences activity of their protein substrates. In Gram-negative bacteria, the process of oxidative folding takes place in the periplasm, whereas in Gram-positive bacteria it occurs in the space between the cytoplasmic membrane and the cell wall [1], [2]. The Dsb system has been studied in detail in *Escherichia coli* K-12 (EcDsb), where it operates in two antagonistic, partially coinciding metabolic pathways, based on the oxidation and the reduction/isomerization reactions [3], [4], [5], [6], [7]. The first reaction (catalyzed by EcDsbA and EcDsbB) appears as the non-selective formation of disulfide bonds in newly synthesized proteins [8], whereas the second (driven by EcDsbC and EcDsbD) ensures the rearrangement of improperly introduced disulfides.

Given the importance of disulfide bond formation to achieve native protein structures, the number of crystallographic studies of DsbA-homologous proteins has risen sharply in the last decade, as reflected by the structures deposited in the Protein Data Bank (PDB) for thirteen of non-redundant, functionally characterized DsbA homologs, ten from Gram-negative and three from Gram-positive bacteria [9]. Despite a common thioredoxin (TRX) fold, members of the DsbA superfamily display numerous structural differences, which result in their various redox properties and substrate specificities, as reviewed by McMahon *et al.* [9]. The delineated differences include, for instance, the sequence of the XX dipeptide within the active-site CXXC motif, which is present in the form of a CPHC in EcDsbA and more than 70% of its homologs [10], [11], [12]. The diverse redox properties of the DsbAs, as well as other TRX-fold proteins, are assumed to be also determined by a residue preceding the CXXC motif and by a residue upstream of the *cis*-Proline loop [13], [14], as well as by indirect interactions of polar residues with the side chain of the N-terminal catalytic cysteine residue [15].

**Figure 1:**
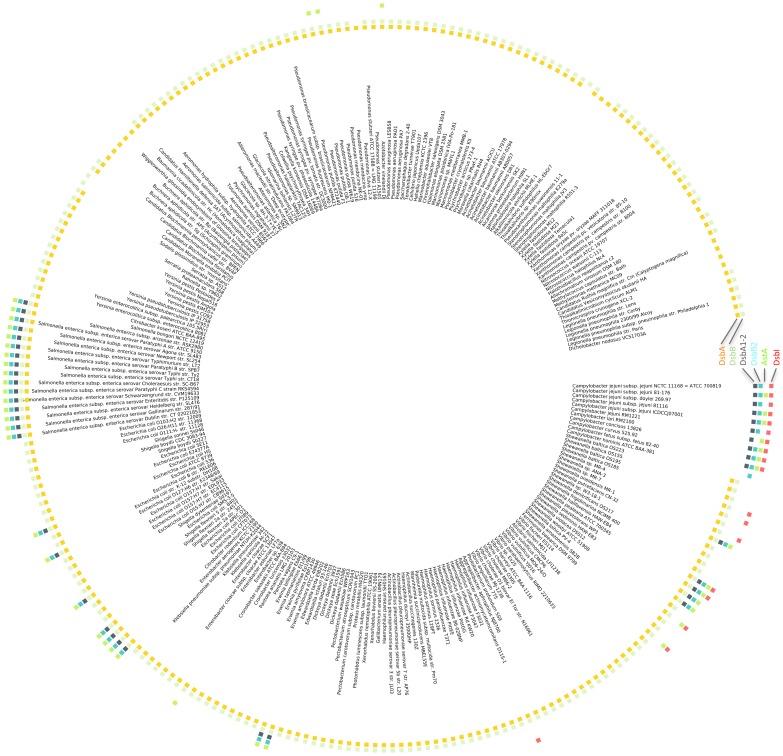
Taxonomic distribution of the DsbA DsbB/I and AstA in gamma-Proteobacteria and epsilon-Proteobacteria. Color boxes indicate the presence of the classical DsbA and DsbB (two inner rings), DsbA1-2, DsbB2, AstA, and DsbI (four outer rings) in a given genome.

Previous reports [16], [17], [18], [19] and our recent updated examination (Figure 1) have revealed that the systems of disulfide bond formation in bacteria are extremely diverse, often involving multiple Dsb homologs and functional analogs. In *E. coli* K-12 two monocistronic units, *dsbA* and *dsbB*, which encode the main oxidative folding enzymes, are located at distinct chromosomal *loci*. Some uropathogenic *E. coli* strains, e. g. UPEC CFT073, encode an additional pair of DsbA-DsbB homologs, namely DsbL-DsbI(DsbB2), that are responsible for the oxidative folding of AstA [20]. These homologs are organized into a three-cistronic operon, *astA-dsbL-dsbI(dsbB2)* in the cited UPEC and *Lellottia amnigena* (formerly *Enterobacter amnigenus*) [21], [22], [23] genomes. Such an additional DsbL-DsbI(DsbB2) pair was also described in *Salmonella enterica* sv. Typhimurium, where the *dsbL-dsbI(dsbB2)* genes are transcribed from a two-cistronic operon, independently of a preceding *astA* gene [24]. The Dsb oxidative pathway of *Campylobacter jejuni* (CjDsb), characterized in this study, seems even more complex and varies between the strains. In the genome of *C. jejuni* strain 81116, it is composed of at least four enzymes, of which two (CjDsbA1 and CjDsbA2) are predicted to localize in the periplasm, and two others (CjDsbB and CjDsbI) – in the inner membrane. The *cjdsb* genes are organized into three operons located at two chromosomal *loci*, i.e. *cjdsbA2-cjdsbB-cjastA* directly followed by a monocistronic unit *cjdsbA1* and a distant *cjdba-cjdsbI*, where *cjastA* codes for the Dsb substrate arylsulfotransferase and *cjdba* codes for the DsbI accessory protein [25]. Such genetic organization suggests a functional analogy of CjDsbA1 to the EcDsbA and CjDsbA2-CjDsbB redox pair to EcDsbL-EcDsbI, however *C. jejuni* 81116 does not possess other EcDsbB homolog that would play analogous role to EcDsbB in re-oxidizing CjDsbA1.

In *C. jejuni*, analogously to *E. coli*, re-oxidation of the EcDsbA homologs, CjDsbA1 and CjDsbA2, is predicted to be achieved by the action of homologs of EcDsbB, CjDsbB and CjDsbI. The classification of the DsbB/DsbI proteins lacks precision. As we have previously shown [25], [26], [27] the name DsbI should be reserved for members of DsbB superfamily that possess five TM helices in the catalytic domain, a CXXC motif located in the periplasmic loop and a beta propeller domain in the C-terminus, as seen in CjDsbI. The proteins named DsbI that are present in some uropathogenic *E. coli* or *S. enterica* proteomes, and which form a redox pair with DsbL, should instead be designated DsbB2 or DsbI(DsbB2) [25], [26].

The process of Dsb oxidative folding of extracytoplasmic proteins is widespread within bacterial kingdom, however as documented by previous studies [16], [17], [18], [19] and our analyses, it demonstrates considerable variability among strains, driven by the horizontal transfer. This work constitutes an attempt to gain insight into the functioning of epsilon-Proteobacterial Dsb systems, which so far have been poorly understood. We studied the *E. coli* DsbA homologs present in *C. jejuni*, CjDsbA1 and CjDsbA2, to unveil their function with respect to oxidative protein folding and to substrate specificity.

## Results

### Phylogeny of Dsb proteins

Classical DsbA and DsbB proteins are present in nearly all gamma-Proteobacteria, but absent in epsilon-Proteobacteria (Figure 1). Sequence analysis of the DsbA family revealed that the DsbA homologs from *C. jejuni*, CjDsbA1 and CjDsbA2, belong to a small cluster named DsbA1-2 that is closely related to the DsbA cluster harboring the classical DsbA proteins, including EcDsbA (Figure S1). For the DsbB family, the DsbB homologs from *C. jejuni*, CjDsbB and CjDsbI, classify to different clusters. CjDsbB belongs to the DsbB2 cluster, which is closely related to the DsbB cluster harboring the classical DsbB proteins, including EcDsbB (Figure S2). CjDsbI, a recently discovered atypical member of the DsbB superfamily [28], localizes within a separate cluster named DsbI, which is distantly related to the two aforementioned DsbB and DsbB2 clusters.

The DsbA1-2 and DsbB2 clusters also contain proteins from several gamma-Proteobacteria, such as *Escherichia* (including EcDsbL and EcDsbI(B2), respectively), *Salmonella*, *Shewanella* and *Shigella*. The presence of DsbA1-2 and DsbB2 cluster members is strongly correlated with each other and with the presence of AstA, a known substrate of the EcDsbL/EcDsbI(B2) system in *E. coli*, but also, to some extent, with the presence of the DsbI-cluster.

To unveil the evolutionary events underlying this taxonomic distribution, we calculated phylogenetic trees for DsbA, DsbB and AstA. We found that DsbA1-2, DsbB2 and AstA have very similar evolutionary histories, suggesting horizontal transfer of the entire DsbA1-2/DsbB2/AstA system from the *Campylobacter* genus to a common ancestor of the gamma-Proteobacterial species, followed by its subsequent loss in most organisms of this clade (Figures S3, S$, and S5). Moreover, the phylogenetic analysis indicates that the divergence of CjDsbA1 and CjDsbA2 occurred after the horizontal transfer event.

CjDsbA1 and CjDsbA2 share a high degree of sequence identity (47%), and their sequence identities to EcDsbA and EcDsbL are 24% and 28% for CjDsbA1 and 28.5% and 39% for CjDsbA2, respectively. The fact that CjDsbA2 is localized in the same transcriptional unit as AstA and DsbB2, and that it is more similar to EcDsbL than CjDsbA1, suggests that it is an evolutionary counterpart of EcDsbL, whereas CjDsbA1 may constitute a later duplication specific for *C. jejuni* strains. This hypothesis is further supported by the fact that some *Campylobacter* genomes, e.g. *C. jejuni* NCTC11168, *C. concisus* 13826 or *C. curvus* 525.92, contain a truncated variant of *cjdsbA2* (encoding a putative TRX-like protein lacking the active CXXC motif) and, at the same time, lack a functional AstA protein.

### Structural modeling of CjDsbA1 and CjDsbA2

**Figure 2:**
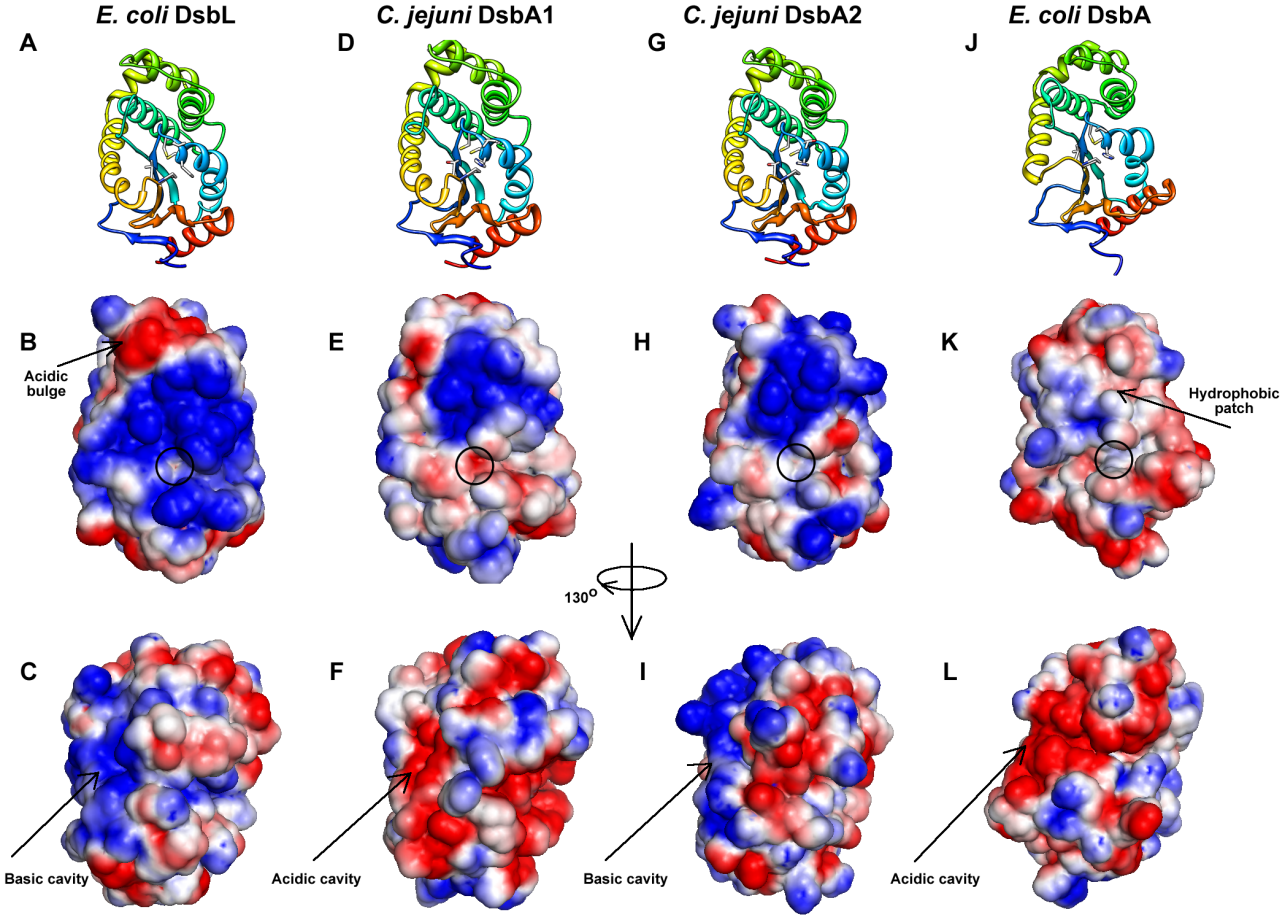
Homology models of *C. jejuni* DsbA1 and DsbA2. *C. jejuni* DsbA1 and DsbA2 (CjDsbA1 and CjDsbA2) models built on *E. coli* DsbA [EcDsbA (PDB ID: 2ZUP [80])] and DsbL [EcDsbL (PDB ID: 3C7M [22])], experimentally characterized members of the DsbA superfamily. Structural representations are shown in ribbon view (A, D, G and J). Electrostatic surfaces coloured by charge from red, acidic, -1kT to blue, basic, +1kT. The orientation in B, E, H and K follows the orientation in the top row (A, D, G and J) and in C, F, I and L is rotated by 130 degrees around the vertical axis, clockwise.

To highlight structural differences between the *C. jejuni* DsbA1 and DsbA2 proteins, we built their homology models based on EcDsbA and EcDsbL structures as templates (Figure 2). Both proteins share an extensive, strongly positively charged electrostatic patch above the active site, as seen for EcDsbL but not for EcDsbA. This finding is in agreement with the phylogenetic analysis and further confirms that CjDsbA1 and CjDsbA2 are more closely related to EcDsbL than to EcDsbA. However, the two proteins differ substantially in charge distribution on the surface that is opposite from the active site. A turn of the model structures of 130° from the active site reveals a surface cavity, which is composed mainly of acidic residues in CjDsbA1 and EcDsbA (Figure 2F and 2L, respectively), in contrast to the basic residues in CjDsbA2 and EcDsbL (Figure 2I and 2C, respectively), suggesting differences in how CjDsbA1 and CjDsbA2 interact with other proteins, e.g. substrates displaying complementary electrostatic charges. This also agrees with the phylogenetic analysis, further supporting the hypothesis that CjDsbA2 is a functional counterpart of EcDsbL.

The differences between CjDsbA1 and CjDsbA2 are also pronounced at the active site. The isoleucine residue in the active CIHC motif of CjDsbA1 is replaced by a threonine residue in the CTHC of CjDsbA2. These CXXC motifs differ from those present in EcDsbA (CPHC) and EcDsbL (CPFC). Also, in both of the CjDsbAs, the so-called *cis*-Pro loop is preceded by a threonine residue (forming a T*c*P motif), as opposed to the valine residue (V*c*P motif) present in EcDsbA and EcDsbL. This could suggest an intermediate character of *C. jejuni* DsbA enzymes between the EcDsbA and EcDsbL oxidoreductases, as well as differences in their respective substrate spectra.

### CjDsbA1 and CjDsbA2 do not promote non-specific aggregation of reduced insulin

**Figure 3:**
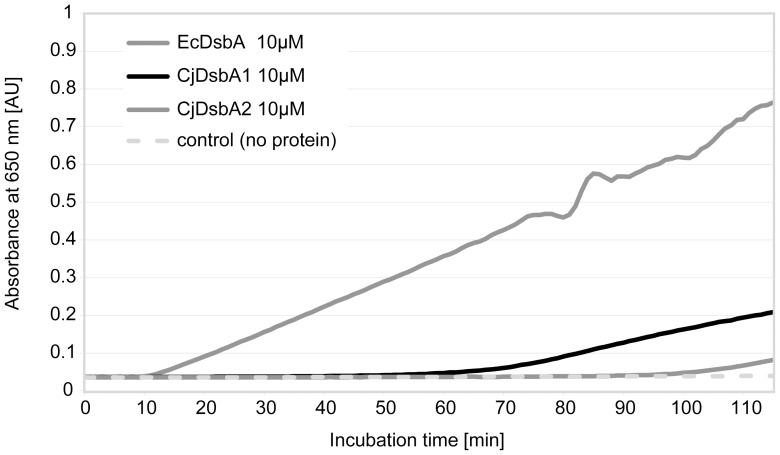
Insulin reduction assay for CjDsbA1 and CjDsbA2. The reaction mixture contained 150 µM insulin in potassium phosphate buffer, pH 7.0 and 2 mM EDTA. The assay was performed in the absence (▪) or presence of 10 µM EcDsbA (♦), CjDsbA1 (⁃) and CjDsbA2 (▴). Reactions started by adding DTT to the final concentration of 0.33 mM and the changes in the absorbance at 650 nm as a function of time were measured. The figure presents a representative result.

Given the predicted structural differences between the two CjDsbAs and their characterized homologs from *E. coli*, we investigated the CjDsbAs disulfide reductase activity in a specific assay, using insulin as a substrate [29], [30]. CjDsbAs catalyzed insulin reduction less efficiently than EcDsbA in the presence of the reductant DTT (dithiothreitol). The onset time of insulin aggregation in this assay (for 0.33 mM DTT) was about 60 min for CjDsbA1 and about 90 min for CjDsbA2, compared to 10 min for EcDsbA (Figure 3).

### CjDsbA1 plays a crucial role in motility and autoagglutination (AAG)

**Figure 4:**
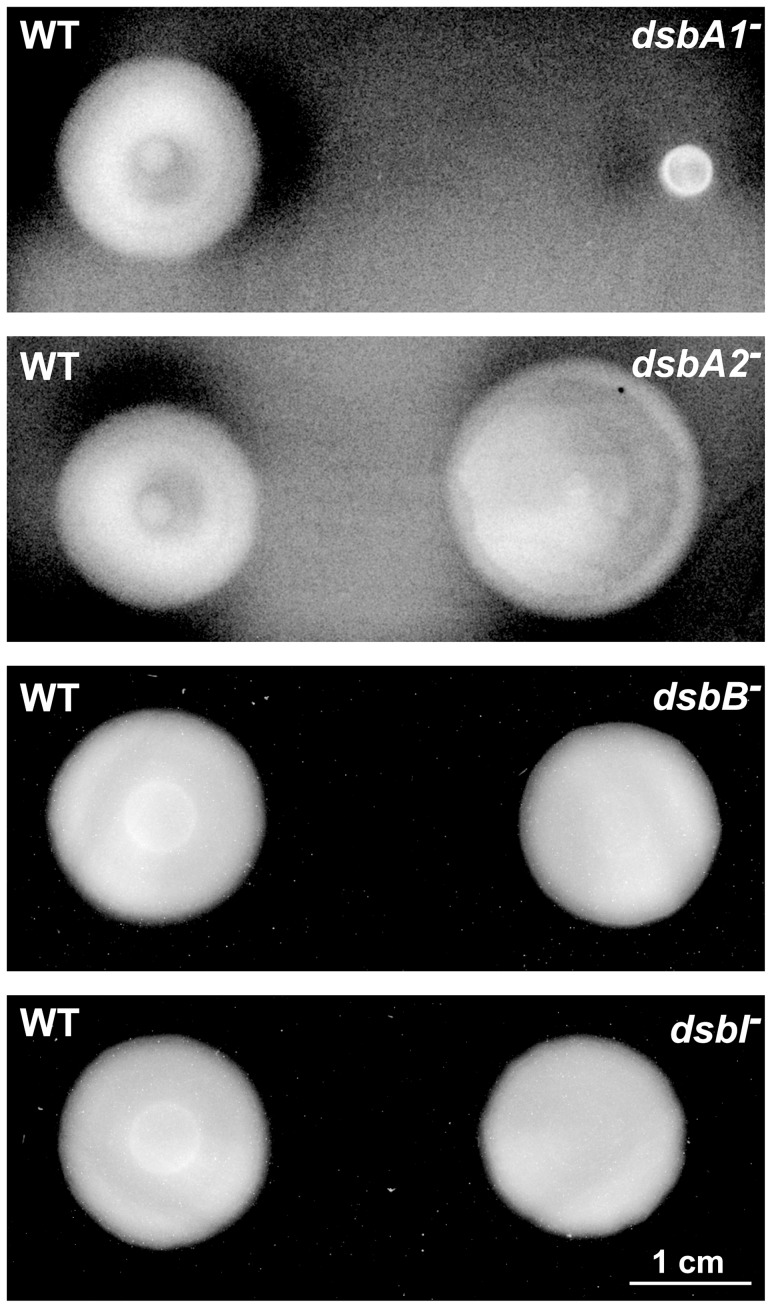
Motility of *C. jejuni* 81116 strains: wild type (WT), *cjdsbA1^-^*, *cjdsbA2^-^*, *cjdsbB^-^* and *cjdsbI^-^* mutants. Bacterial motility was monitored after 24 hours of incubation on 0.4% MH-agar plates. The *cjdsbA1^-^* strain is non-motile, contrary to the wild type (WT), *cjdsbA2^-^*, *cjdsbB^-^* and *cjdsbI^-^* strains.

To assess the role of the two CjDsbAs in *C. jejuni* motility, we performed soft agar growth assays for *cjdsb* mutant strains. We found that the *C. jejuni dsbA1* mutant was unable to move beyond the stab point of inoculation on soft agar, whereas the *C. jejuni* wild type and the *cjdsbA2* mutant were both motile, as were the *dsbB* and *dsbI* mutants (Figure 4).

**Figure 5:**
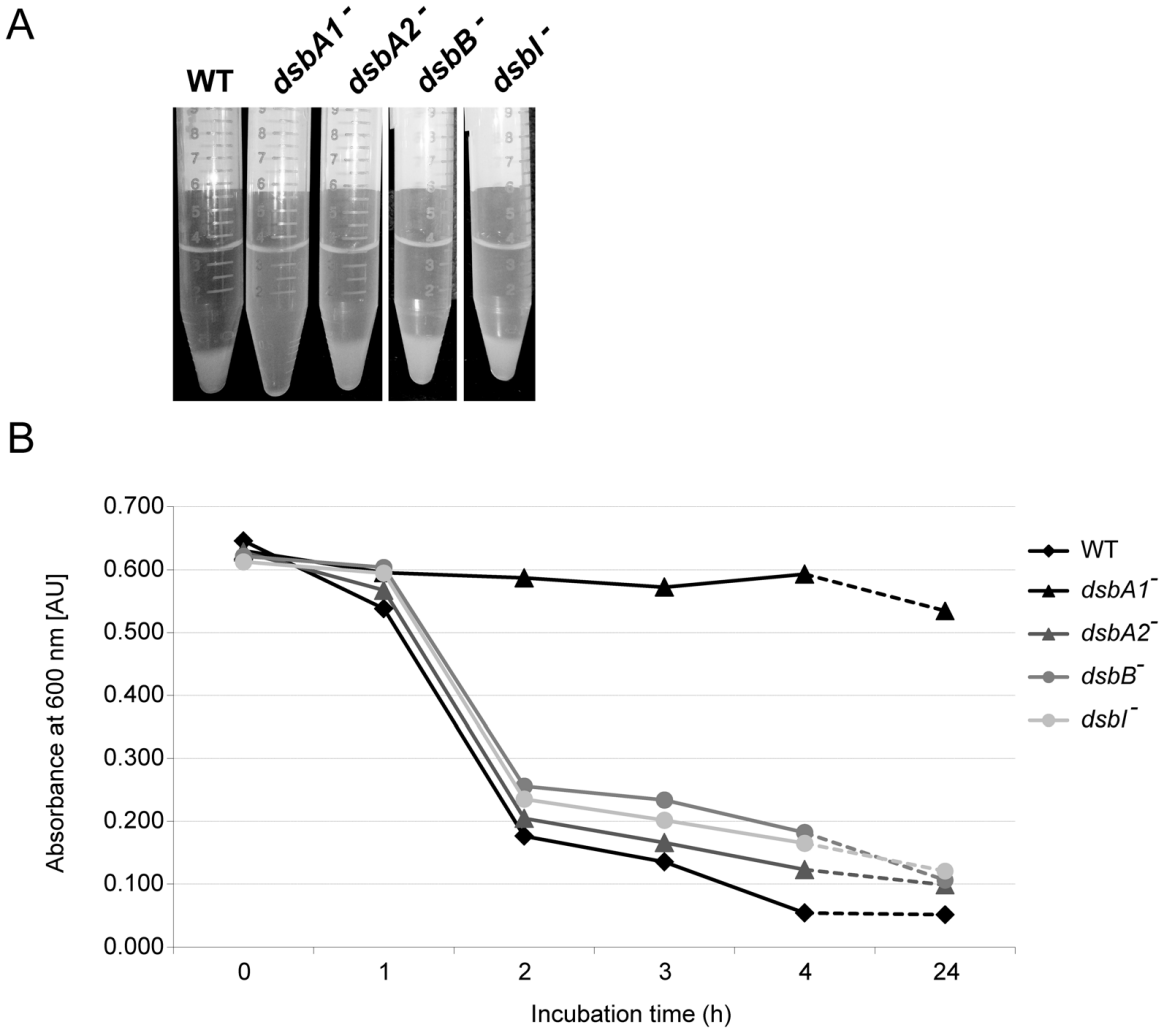
Autoagglutination of *C. jejuni* 81116 strains: wild type (WT), *cjdsbA1^-^*, *cjdsbA2^-^*, *cjdsbB^-^* and *cjdsbI^-^* mutants. Bacterial autoagglutination was monitored as a decrement of turbidity (A) or optical density (B) of bacterial suspension in LB at room temperature after harvesting cells from BA plates. The *cjdsbA1^-^* strain does not autoagglutinate, contrary to the wild type (WT), *cjdsbA2^-^*, *cjdsbB^-^* and *cjdsbI^-^* strains. The figure presents a representative result.

The impact of Dsb system on *C. jejuni* autoagglutination was established by an aggregation assay. When bacterial cultures were incubated without shaking at room temperature, the *C. jejuni dsbA1* mutant failed to autoagglutinate, and its cells remained in suspension, whereas the wild type *C. jejuni* and the *cjdsbA2* mutant both autoagglutinated and formed clumps, as did the *dsbB* and *dsbI* mutants (Figure 5).

These observations were confirmed when the strains for motility and aggregation assay were cultured on defined F12 medium, which does not contain cystine and thus prevents non-specific oxidation of CjDsbA1. The lack of CjDsbB did not influence neither motility nor autoagglutination (Figure S6).

### CjDsbA2 assists the oxidative folding of *C. jejuni* arylsulfotransferase AstA

Arylsulfotransferase, which is encoded in an operon with the *C. jejuni dsbA2* and *dsbB* genes (*cjdsbA2-cjdsbB-cjastA*), plays a role in detoxification of phenolic compounds [31], [32], catalyzing a sulfuryl transfer from a phenolic sulfate to a phenol, *via* a Ping-Pong mechanism that differs from the PAPS (3′-phosphoadenosine 5′-phosphosulfate)-dependent mammalian sulfotransferases [33], [34]. In *E. coli* cells, arylsulfotransferase functions as a homodimer, containing single disulfide bond that confers enzyme activity, Cys445-Cys451 (C2–C3) [33]. *C. jejuni* AstA possesses four cysteine residues, namely Cys8, Cys346, Cys449, Cys456; the first cysteine is not conserved and is located within the signal peptide, but the three others are well conserved in the primary structures of their close homologs (AstA from *E. coli, Salmonella enterica* sv. Typhimurium and *L. amnigena*).

**Figure 6:**
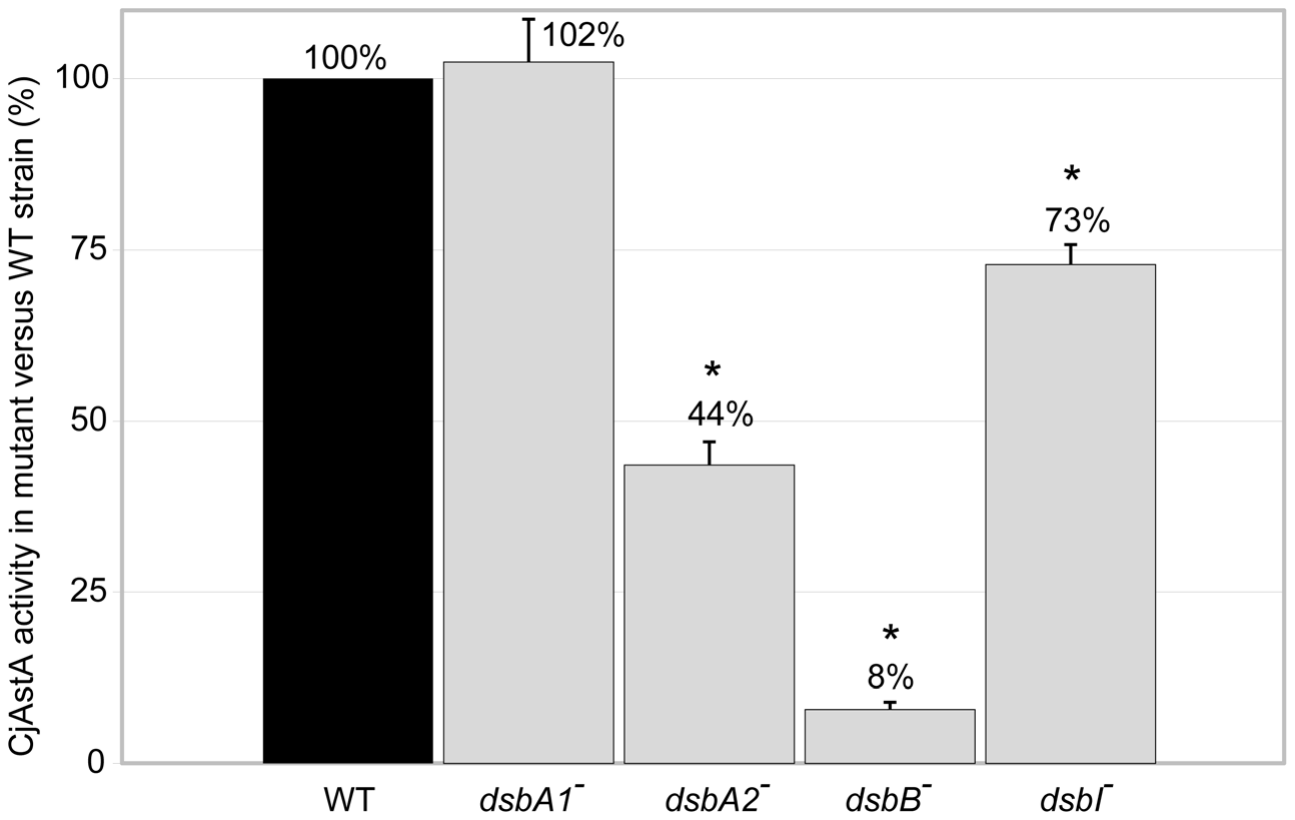
Arylsulfotransferase AstA activity in *C. jejuni* 81116 strains: wild type (WT), *cjdsbA1^-^*, *cjdsbA2^-^, cjdsbB^-^* and *cjdsbI^-^* mutants. The diagrams illustrate mean values and standard deviations of AstA activity derived from three experiments; for each experiment the AstA activity were carried out in triplicate. Statistical significance was calculated using Student *t* test for comparison of independent groups (GraphPad Prism) with reference to the AstA activity in the wild type (WT) strain. P values of P<0.05 were considered statistically significant (*).

Given that *cjastA* localizes in the *C. jejuni* chromosome between the *cjdsbA2-cjdsbB* and *cjdsbA1* loci and forms a transcriptional unit with the preceding *cjdsbA2* and *cjdsbB*, we hypothesized that its activity is also dependent on oxidative folding carried out by these oxidoreductases. To assess the direct impact of each of the CjDsb enzymes on CjAstA activity, we performed qualitative and quantitative AstA assays. In the qualitative AstA assay, *C. jejuni* cells were grown on MH supplemented with XS (5-bromo-4-chloro-3-indolylsulfate, a chromogenic substrate of AstA that is hydrolyzed to blue-colored 5-bromo-4-chloro-indol). On this media, the *cjdsbA*1 and the *cjdsbI* mutants, as well as the wild type strain, formed blue colonies; whereas the *cjdsbA2* mutant formed white colonies with blue shadow and the *cjdsbB* mutant formed white colonies. The *C. jejuni* NCTC11168 strain, which possesses a truncated version of *cjdsbA2* (coding for a putative DsbA2 protein lacking the CXXC motif) and an *astA* pseudogene, was employed as a negative control; it produced white colonies (data not shown). These observations indicated a reduced CjAstA activity in the *cjdsbA2* strain and the lack of any AstA activity in *cjdsbB* mutant, as compared to the wild type strain. These results were confirmed by a quantitative AstA activity assay. The AstA activity in the *cjdsbA1* mutant was comparable to the level observed for the wild type strain (102%), whereas in the *cjdsbA2* mutant, it was reduced to 44%. In the *C*. *jejuni* 81116 *dsbB* and *dsbI* mutant strains, the AstA activity reached respectively only 8% and 73% of the activity observed for the wild type cells (Figure 6). These results confirm that efficient oxidative folding is critical for *C. jejuni* AstA activity and that the periplasmic CjDsbA2 plays a crucial role in this process, in cooperation with the membrane oxidoreductase CjDsbB; in contrast, CjDsbA1 and CjDsbI are not essential for CjAstA oxidative folding and activity.

### CjDsbA1 assists the oxidative folding of C. jejuni alkaline phosphatase PhoX

**Figure 7:**
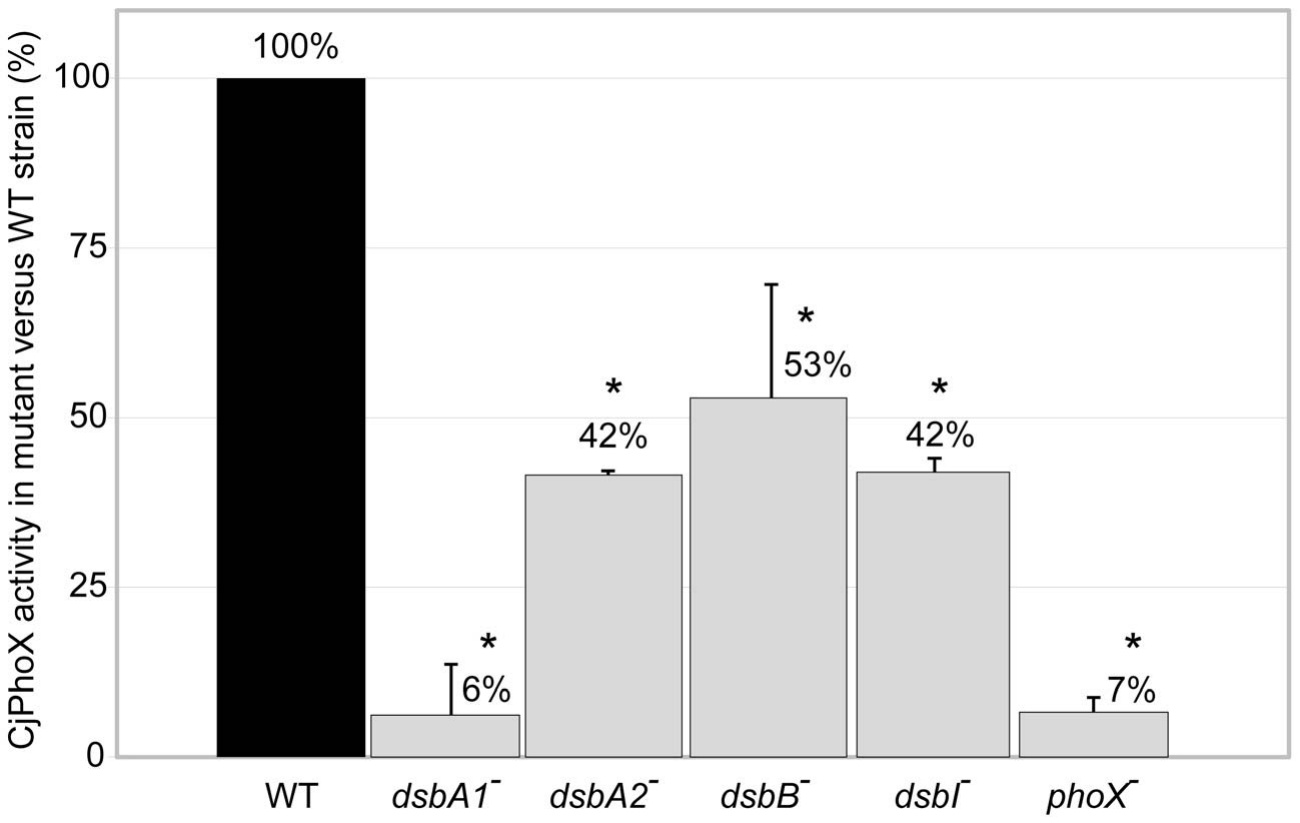
Alkaline phosphatase PhoX activity in *C. jejuni* 81116 strains: wild type (WT), *cjdsbA1^-^*, *cjdsbA2^-^, cjdsbB^-^* and *cjdsbI^-^* mutants. The diagrams illustrate mean values and standard deviations of PhoX activity derived from three experiments; for each experiment the PhoX activity were carried out in triplicate. Statistical significance was calculated using Student t test for comparison of independent groups (GraphPad Prism) with reference to the PhoX activity in the wild type (WT) strain. P values of P<0.05 were considered statistically significant (*).

Alkaline phosphatase CjPhoX, encoded at a distant chromosomal *locus* from any of the *C. jejuni dsb* genes, provides bacterial cells with a phosphorous source. *E. coli* PhoA homologs use phosphomono- and phosphodiester substrates, with Ca^2+^ or Mg^2+^ as a cofactor. PhoA functions in the periplasmic space of *E. coli* as a homodimer, containing two disulfide bonds with different roles; Cys286-Cys336 (C3–C4), which is sufficient for native protein activity, and Cys168–Cys178 (C1–C2), which does not affect native protein activity but is required for a protease-resistant, stable enzyme structure [35]. CjPhoX, like the alkaline phosphatases from several other bacteria, i.e., *Pseudomonas aeruginosa* and *Vibrio cholerae*, is an atypical functional analogue of *E. coli* PhoA (EcPhoA), as it exclusively uses phosphomonoester substrates and requires Ca^2+^ as the cofactor. It is regulated by a two-component system, PhoS/PhoR, and is secreted *via* the Tat pathway [36]. The *C. jejuni* PhoX possesses five cysteine residues, namely Cys198, Cys211, Cys399, Cys519, Cys540, that are all conserved in the primary structures of its closest homologs (PhoX from *V. cholerae* and from *P. aeruginosa*). Disulfide connectivity predictions performed using the DiANNA server suggested that four of the cysteine residues, Cys198–Cys519 (C1–C4) or Cys211–Cys540 (C2–C5), might be involved in forming disulfide bonds [37]. Given the atypical character of the *C. jejuni* functional analogue of the EcPhoA enzyme, we decided to analyze whether the cysteine residues confer enzyme activity for CjPhoX. To test this, we constructed plasmid-encoded point-mutant versions of CjPhoX, in which a single cysteine residue was replaced with alanine (see Methods section for details), and we introduced them into the cells of the *cjphoX* mutant. Then we performed an alkaline phosphatase enzymatic assay for each strain. Changing C198A, C399A and C540A did not alter the PhoX activity, whereas the C211A and C519A mutations led to a significantly reduced PhoX activity (to 5% and 50%, respectively) in comparison to the wild type enzyme. These results (Figure S7) indicated that there is only one disulfide bond in CjPhoX that affects enzyme activity, and that bond is formed between Cys211–Cys519 (C2–C4). In a quantitative PhoX activity test, we found that in the *C. jejuni* 81116 *cjdsbA1* it reached only 6% of the activity observed for wild type cells, whereas in the *cjdsbA2* and *cjdsbI* mutant strains it was reduced to 42% and in the *cjdsbB* mutant to 53% of the wild type activity. The *C. jejuni* 81116 *phoX* mutant strain, employed as a negative control, produced 7% of the wild type activity (Figure 7).

### CjDsbA1 can cooperate with EcDsbB and CjDsbB

The results of AstA activity assay clearly showed that CjDsbA2 forms the redox pair with the CjDsbB that is encoded in the same operon. Subsequently, we focused on revealing the cooperation between DsbA1 and DsbB in *C. jejuni* network functioning. We attempted this in two ways: first, by comparing the CjDsbA1 redox state in various *dsb* mutants to the redox state observed for the wild type strain; and second, by testing the ability of CjDsbA1 to complement *E. coli dsbA* and *dsbA dsbB* mutants.

**Figure 8:**
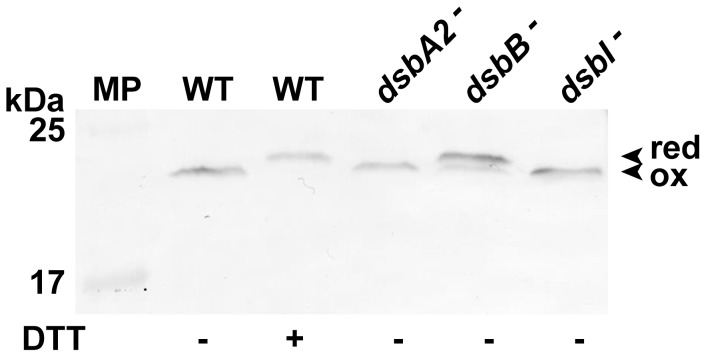
Redox state of CjDsbA1 in *C. jejuni* 81116 strains: wild type (WT), *cjdsbA2^-^*, *cjdsbB^-^* and *cjdsbI^-^* mutants. Bacterial cultures were treated with 10% TCA, followed by alkylation with AMS (4-acetamido-4′-maleimidylstilbene-2,2′-disulfonic acid). Cellular proteins including the reduced (red; DTT treated, modified by AMS) controls were separated by 15% SDS-PAGE under non-reducing conditions, followed by Western blot analysis using antibodies against CjDsbA1. Each lane contains proteins isolated from the same amount of bacteria. The relative positions of protein molecular weight standard (MP) are listed on the left (in kilodaltons). The figure presents a representative result.

As specific anti-CjDsbA1 serum does not exhibit cross reactivity with CjDsbA2 (unpublished data), we used it to determine the CjDsbA1 redox state *in vivo* in the *C. jejuni* 81116 wild type strain and in its isogenic *dsb* mutants, employing the AMS -trapping technique, which distinguishes reduced and oxidized dithiols [38], [39]. The results are presented in Figure 8. Consistent with the previous results, we found that the CjDsbA1 is present in the oxidized form in wild type *C. jejuni* 81116, which confirmed the oxidizing activity of CjDsbA1 in *C. jejuni*. The absence of CjDsbB resulted in the presence of CjDsbA1 in the reduced form, whereas the absence of the CjDsbI membrane oxidoreductase did not influence the redox state of CjDsbA1. These results indicate that CjDsbA1 is maintained in an oxidized state, at least partially, by CjDsbB and not by CjDsbI.

**Figure 9:**
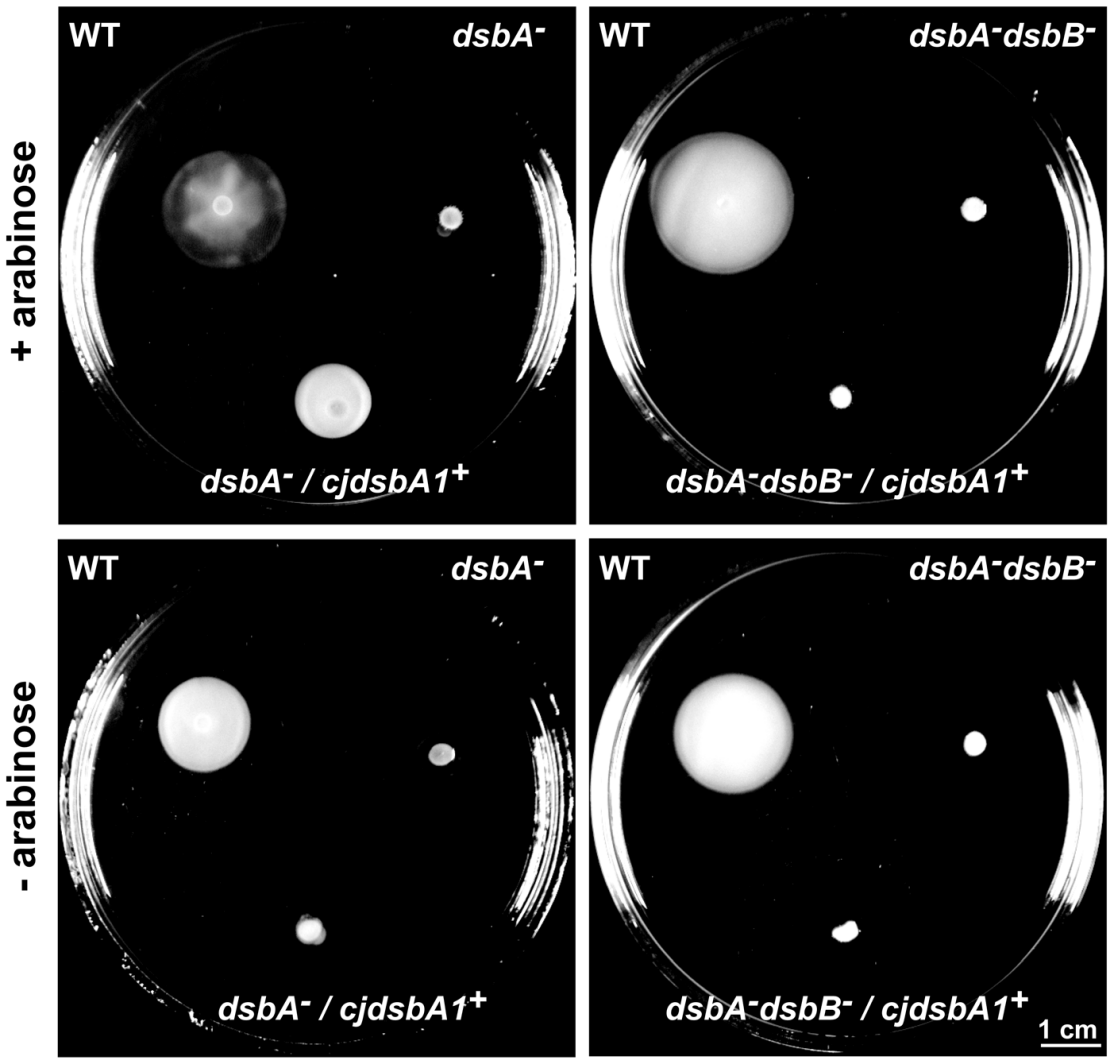
Complementation of *E. coli dsbA^-^ and dsbA^-^ dsbB^-^ mutants by CjDsbA1*. Bacterial motility was monitored after 24 hours of incubation on 0.35% MH-agar plates with (upper panel) or without (lower panel) arabinose induction of CjDsbA1. The *E. coli dsbA^-^* and the double *dsbA^-^ dsbB^-^* strains are non-motile, contrary to the *E. coli* wild type (WT) and *dsbA^-^* complemented *in trans* with *cjdsbA1*, whereas *E. coli dsbA^-^ dsbB^-^* complemented *in trans* with *cjdsbA1* remains non-motile. The figure presents a representative result.

The ability of CjDsbA1 to complement a DsbA deficiency in *E. coli* was assessed using the recovery of cell motility as a phenotypic trait. The *cjdsbA1* gene was expressed in an *E. coli dsbA* mutant while under control of an arabinose inducible promoter cloned in a low-copy number plasmid. Expression of the protein was confirmed by Western blot, using specific polyclonal rabbit anti-CjDsbA1 antibody (data not shown). We first tested whether the expression of *cjdsbA1* in the *E. coli dsbA* mutant restored cell motility. Next, in order to assess whether the activity of CjDsbA1 in *E. coli* was dependent on the presence of *E*. *coli* DsbB, we introduced a plasmid-encoded CjDsbA1 into *E. coli* JCB818 cells (*dsbA dsbB* double mutant). As shown in Figure 9, CjDsbA1 complements the EcDsbA deficiency in an EcDsbB-dependent manner.

Taken together, our results indicate that CjDsbA1 is re-oxidized by EcDsbB and only partially by CjDsbB. However, in its native host, CjDsbA1 can also be re-oxidized by other proteins, as CjDsbB is not essential for cell motility, autoagglutination and PhoX activity.

## Discussion

The process of the oxidative folding of bacterial extracytoplasmic proteins through introduction of disulfide bonds displays significant diversity within the bacterial kingdom [10], [40]. Most *Campylobacter jejuni* strains contain two functional DsbA oxidoreductases (CjDsbA1 and CjDsbA2), which are close homologs of DsbL. Our previous studies on the expression of *C. jejuni dsb* genes revealed a higher level of expression of the monocistronic transcriptional unit – the *cjdsbA1* gene (*c8j_0814* in *C. jejuni* 81116) – than the tri-cistronic operon comprised of *dsbA2-dsbB-astA* (*c8j_0811-0812-0813* in *C. jejuni* 81116) [25].

The predicted overall structure of both CjDsbA1 and CjDsbA2 is similar to that of EcDsbA and EcDsbL. The two CjDsbAs share an extensive, strongly positively charged electrostatic patch above the active site, as seen for *E. coli* DsbL but not for *E. coli* DsbA. Noticeable differences between the two CjDsbAs and the majority of DsbAs or DsbLs occur in the CXXC active-site motif, as well as in the residue preceding the *cis*-Pro motif. Both motifs are responsible for numerous physico-chemical properties of these enzymes and play a significant role in their folding and stability, as well as in the interaction of DsbA with its redox partner(s) (i.e., DsbB) and substrates [11], [14], [41], [42]. These motifs are most frequently found in classical EcDsbA and EcDsbL homologs as CPHC and CPFC, and as V*c*P, respectively [10], [11]. However, in CjDsbA1 and CjDsbA2 they are represented by CIHC and CTHC, and by T*c*P, respectively. The motifs found in the CjDsbAs are extremely rare, they are present in only six among the bacterial DsbA homologs so far identified by *in silico* analysis [10]. Interestingly, the presence of a threonine residue in the *cis*-Pro loop of CjDsbA1 and CjDsbA2 is characteristic for the dimeric EcDsbC and EcDsbG oxidoreductases that are involved in the reduction/isomerization pathway of extracytoplasmic protein oxidative folding.

*C. jejuni* DsbA1 and DsbA2 differ significantly with respect to the distribution of electrostatic potentials on the surface that is distant from the active site. In this respect, CjDsbA1 resembles EcDsbA, whereas CjDsbA2 is similar to EcDsbL. These differences are reflected in a lack of immunological similarity between the two CjDsbAs, since anti-rCjDsbA1 antiserum does not recognize CjDsbA2.

These structural differences between CjDsbA1 and CjDsbA2 may indicate that they have different selectivity for interactions with other proteins. The *in vitro* insulin reduction assay demonstrated that both CjDsbAs are less active in insulin reduction than EcDsbA what may result from strongly positively charged electrostatic patches above the active site of the CjDsbAs, also observed for EcDsbL [20]. Additionally, the long onset time of insulin aggregation by CjDsbA2 may result from the presence of the basic residues on its surface opposite from the active site.

Taking into consideration the previously reported *C. jejuni dsb* gene expression regulation [25], as well as the functional, phylogenetic and structural analyses of the two CjDsbAs presented in this work, CjDsbA1 seems to be the primary Dsb oxidase in *C. jejuni*, whereas CjDsbA2, together with CjDsbB, is a counterpart of EcDsbL-EcDsbI(DsbB2) system that is specific for AstA. Considering the fact that *C. jejuni* lacks the classical, broad spectrum DsbA-DsbB system, it is plausible that CjDsbA1 partially replaces it. This hypothesis is supported further by the observation that loss of CjDsbA1 results in the loss of motility and autoagglutination, two processes that are important for bacterial virulence. Our results show that both motility and autoagglutination are dependent on CjDsbA1, and not on CjDsbA2. In *E. coli,* DsbA is critical for motility at the step of flagella assembly, as FlgI – the flagella P-ring protein – is the substrate for DsbA [43]. An examination of the CjFlgI (C8J_1368 in *C*. *jejuni* 81116 genome) amino-acid sequence revealed that this protein does not contain cysteine residues, and thus it cannot be a Dsb system substrate. Instead, there is another *C. jejuni* protein involved in the process of flagellum biogenesis, namely CjFlhA (C8J_0820 in *C*. *jejuni* 81116 genome), that contains four cysteine residues in its primary structure and is thus a potential target of CjDsb system [44], contrary to its homolog in *E. coli* genome, EcFlhA, which does not possess any cysteine residue. CjFlhA is engaged in the protein transport system that translocates proteins that are participating in flagellum biogenesis and components of the type III transport system. It was reported that *cjflhA* mutant strains are devoid of flagella and are seriously impaired with respect to chicken colonization, invasion and autoagglutination [45]. Disulfide connectivity predictions performed using the DiANNA server suggested that four of the CjFlhA cysteine residues, Cys35–Cys136 (C1–C2) and Cys680–Cys693 (C3–C4), might be involved in forming disulfide bonds [37]. Further studies are necessary to confirm the hypothesis that Dsb proteins influence the CjFlhA protein fold.

As revealed by the CjAstA activity assays, the presence of CjDsbA2 oxidoreductase is indispensable for oxidative folding of cellular CjAstA. Lack of chromosomally encoded CjDsbA1 did not impact the AstA activity. Analogous results were observed for uropathogenic strains of *E. coli* UPEC CFT 073 and *S. enterica* sv. Typhimurium. In UPEC CFT073, the EcDsbL-EcDsbI(B2) redox pair rather than the EcDsbA/EcDsbB redox pair, is mainly responsible for AstA activity [20]; in *S. enterica* sv. Typhimurium, out of the three DsbAs (i.e., SeDsbA, SeDsbL and SeSrgA), only SeDsbL-SeDsbI(B2) redox pair restored the full activity of AstA in a triple *dsbA*-homolog mutant [24].

We previously documented the operon organization of the *cjdsbA2-cjdsbB-cjastA* chromosomal locus [25]. Similar to UPEC CFT 073 and *S. enterica* sv. Typhimurium, the *astA-dsbL-dsbI(B2)* genes localize in the same chromosomal locus, but in UPEC they are organized in a tri-cistronic operon, and in *S. enterica,* the *astA* gene is transcribed independently and the *dsbL-dsbI(B2*) forms a two-cistronic transcriptional unit [24]. Given all these arguments, we suggest that CjDsbA2 and CjDsbB, encoded in the same operon, form a functional redox pair responsible for the introduction of the disulfide bond that conditions CjAstA activity, a hypothesis that is reinforced by the complete abolition of CjAstA activity in a *cjdsbB* mutant. Partial activity of CjAstA in the *cjdsbA2* mutant, as observed for both the qualitative and quantitative tests, leads us to propose the existence of another, yet unidentified protein present in the *C. jejuni* periplasm that is a functional analog of CjDsbA2 and also cooperates with CjDsbB. Further biochemical tests indicated that CjDsbA1 catalyzes the formation of disulfide bonds in CjPhoX, a functional analog of the EcPhoA that is a classical substrate for the DsbA-DsbB redox pair in *E. coli* K-12 [30]. For comparison, the *E. coli dsbA* knock-out strain displayed 30% of the PhoA activity observed for the wild type *E. coli* strain, as has been documented elsewhere [46]. Chromosomally encoded CjDsbA2 does not participate in this process, which supports the hypothesis of the specificity of CjDsbA2 for its periplasmic substrate, CjAstA.

To confirm that the observed phenotypic changes resulted from the lack of CjDsbA1, we conducted a complementation experiment. The plasmid-carrying wild type version of *dsbA1* gene (see Methods section for details) was introduced into a *C. jejuni* strain in which the chromosomal *dsbA1* gene was deleted. The motility test served as a phenotypic trait to track DsbA1 activity. We found that the loss of motility in *C. jejuni dsbA1* ^-^ was not restored by the plasmid cj*dsbA1* version, meaning that plasmid-encoded CjDsbA1 delivered *in trans* failed to complement the *C. jejuni dsbA1* mutation. We conclude that overproduction of the CjDsbA1 disturbed the balance among oxidoreductases functioning in the *C. jejuni* cells. Similar results for *E. coli* were reported [47], [48], where the complementation of an EcDsbA deficiency by mutated variants of thioredoxin was observed only when they were expressed at low level. Similarly, Kadokura *et al.* used pBR322 plasmids with weakened *lac* or *trc* promoters to study the effect of a *cis*-Pro loop preceding residue on capacity of EcDsbA to oxidize substrate proteins [42].

Our data clearly documented that CjDsbA2 forms a redox pair with CjDsbB and is involved in AstA oxidative folding. However, the cooperation between CjDsbA1 and CjDsbB is not clear. Redox experiments demonstrated that CjDsbB is at least partially responsible for CjDsbA1 re-oxidation, given that CjDsbA1, which is present in the oxidized form in wild type cells, was detected in both the oxidized and reduced forms in a *cjdsbB* mutant. Additionally, *cjdsbA1* introduced *in trans* into an *E. coli dsbA* mutant restores the ability of the bacteria to swarm on the top of a soft agar plate in a DsbB-dependent manner. However, at the same time we found that the oxidation of CjPhoX is not fully abolished in *dsbB-* or *dsbI-*mutated strains, and both these strains are motile, which contrasts with the *cjdsbA1* mutant. Such inconsistencies in experimental data may suggest that CjDsbA1 could be oxidized by an additional, as yet unidentified, oxidoreductase(s), as these phenomena cannot be explained by non-specific oxygen- or cysteine-dependent formation of disulfide bonds. This hypothesis is also based on the findings of our preliminary comparison of the periplasmic subproteome of a double-mutant *C. jejuni dsbB dsbI* strain with the wild type strain (data not shown). The inactivation of the two membrane oxidoreductases, i.e., CjDsbB and CjDsbI, resulted in an increased level of enzymes such as thioredoxin (TRX) and thioredoxin reductase, which appear in the periplasmic cell fraction of the double mutant (data not shown). Given the periplasmic protein fractions were not contaminated with cytoplasmic proteins as verified by means of the isocitrate dehydrogenase (ICDH) assay, described by *et al*. (1991) [49] and Myers *et al*. (2005) [50], this finding supports the potential involvement of TRX in protein oxidative folding. The involvement of TRX agrees with the observation that the *in vivo* redox function of some TRX-fold proteins depends on their structure and localization, and therefore, they have an intrinsic ability to switch between the antagonistic reactions: from reduction to oxidation. As an example, the *E. coli* TRX (encoded by *trxA*) can switch its activity from a monomeric disulfide reductase to an oxidase upon being artificially engineered into a dimeric protein, but also upon being transported from the cytoplasm to the periplasm by the simple addition of an N-terminal signal sequence [48], [51], [52]. Moreover, Shu-Sin Chng *et al.* (2012) recently reported that overexpressing PspF, a periplasmic rhodanase enzyme containing only one cysteine residue, partially restores disulfide bond formation in an *E. coli dsbA* mutant, in an EcDsbC-dependent, but not an EcDsbB-dependent, manner [53]. An alternative explanation of these observations could be the increased permeability of the *C. jejuni* outer membrane caused by the absence of CjDsbB. This explanation is consistent with a phenomenon observed in previous studies of two *E. coli* mutants: a double mutant, *dsbA dsbC,* and a mutant lacking the OmpL porin [46], [54].

## Conclusions

In this work we studied detailed functioning of two paralogous CjDsbA proteins, CjDsbA1 and CjDsbA2, from *C. jejuni* 81116. Our results demonstrated not only differences in their substrate spectra and functional redundancy in their oxidative protein folding systems, but also their intricate evolutionary history. However, CjDsbA1 appears to be the primary oxidative folding catalyst in *C. jejuni* cells, whereas the importance of CjDsbA2 is secondary. We also found evidence pointing to an additional, as yet unidentified, periplasmic oxidoreductase(s) or an entire pathway, which might play a role in *C. jejuni* protein oxidative folding, since CjDsbA1 remains active in the absence of CjDsbB. At this stage we could propose the simple preliminary model of the Dsb proteins functioning in oxidative protein folding in *C. jejuni* cells (Figure S8), relating to those given by Heras *et al.* [10], however its confirmation would require further studies. Moreover, our phylogenetic analysis indicates that the common ancestor of the CjDsbAs and other epsilon-Proteobacterial DsbAs gave rise to the DsbL lineage, which includes the well-characterized EcDsbL, *via* horizontal transfer to gamma-Proteobacteria. CjAstA and CjPhoX are the first Dsb-dependent enzymes ever identified in *C. jejuni*.

## Methods

### Bioinformatics analyses

Sequence searches on the NCBI protein database were conducted with BLAST [55] with sequences of known DsbA family members (gi: 218562494, 218562492, 26249617, 26250621) and DsbB family members (gi: 121613190, 121613521, 161486244, 26249618) as queries. The sequences obtained that belonged to the DsbA and DsbB families were independently classified into clusters using CLANS [56]. Sequences originating from canonical DsbA and DsbA1–2 clusters were grouped together, aligned using MUSCLE [57], filtered to 90% pairwise similarity using hhfilter [58] and used to construct a phylogenetic tree with Fasttree2 [59]. The phylogenetic tree of the DsbB and DsbB2 clusters members were constructed with the same procedure.

### Protein structure prediction and analysis

*C. jejuni* DsbA1 and DsbA2 amino acid sequences were submitted to the GeneSilico metaserver gateway [60] for secondary structure prediction and fold recognition. *E. coli* DsbA (PDB ID: 1FVK [61]) and *E. coli* DsbL (PDB ID: 3C7M [22]) have been identified as the best templates for homology modeling. The modeling followed the “Frankenstein's monster” approach [62] of iterative modeling with MODELLER [63], model evaluation by MetaMQAP [64] and recombination of alignments corresponding to best-scored parts of models. Molecular surface and charge distribution were generated using CHARMM-GUI [65] and visualized with PyMOL [66]. Structural representations were created with UCSF CHIMERA [67].

### Bacterial strains, plasmids, media and culture conditions

All strains and plasmids used in this study are listed in Table 1.

**Table 1:** Bacterial strains and plasmids used in this study.

Strain/Plasmid	Genotype or relevant characteristics	Origin
***C. jejuni*** ** strains**		
81116	parental strain	[81]
81116 *dsbA1*	*dsbA1*::*aphA3*	This study
81116 *dsbA2*	*dsbA2*::*cat*	This study
81116 *dsbB*	*dsbB*::*aphA3*	This study
81116 *dsbI*	*dsbI*::*cat*	This study
81116 *phoX*	*phoX*::*aphA3*	[71]
***E. coli*** ** strains**		
DH5α	F^-^ Φ80*lac*ZΔM15 Δ(*lacZYA-argF*) U169 *rec*A1 *endA1 hsdR17* (rK–, mK+) *phoA supE44* λ– *thi*-*1 gyrA96 relA1*	[70]
TG1	*supE44 hsd*Δ5 *thi* Δ(*lac- proAB*) F' [*traD36 proAB* ^+^ *lacI^q^ lacZ*ΔM15]	[70]
JCB817	MC1000 *phoR* λ102 *dsbA::kan1*	[30]
JCB818	MC1000 *phoR* λ102 *dsbA::kan1 dsbB::kan*	[30]
**General cloning and expression vectors**		
pGEM-T Easy	3 kbp; Ap^r^; *ori* ColE1; LacZα	Promega
pBluescript II SK	3 kbp; Ap^r^; *ori* ColE1; LacZα	Stratagene
pET22b	5.53 kbp; Ap^r^; *ori* ColE1; P*_lac_*	Novagen
pBF14	12.1 kbp; Km^r^; *ori* ColE1	J. van Putten
pRY109	3.5 kbp; Cm^r^; *ori* ColE1	[82]
pMPM-A6	6.85 kbp; Ap^r^, Sp^r^; *ori* p15A; P*_araBAD_*	[72]
**Plasmids for recombinant CjDsbA1, CjDsbA2 and EcDsbA overexpression and purification**		
pUWM837	pET22b / *ss*'*pelB-cjdsbA1-his_6_*	This study
pUWM1065	pET22b / *ss*'*pelB-cjdsbA2-his_6_*	This study
pDEST14-*ecdsbA*	pDEST14-*ecdsbA-his_6_*	J.F. Collet
**Plasmids for complementation experiments**		
pUWM1213	pGEM-T Easy / *cjdsbA1*	[28]
pUWM1214	pRY111 / *cjdsbA1*	[28]
pUWM1246	pMPM-A6 / *ss*'*pelB-cjdsbA1-his_6_*	This study
**Plasmids for mutagenesis**		
pUWM607	pGEM-T Easy / *cjdsbB::aphA3*	[28]
pUWM713	pGEM-T Easy / *cjdsbI::cat*	[25]
pUWM825	pBluescript II SK / *cjdsbA2::cat*	This study
pUWM1306	pGEM-T Easy / *cjdsbA1::aphA3*	This study
**Plasmids for ** ***cjphoX*** ** site-directed mutagenesis**		
pMA1-*cjphoX*	pMA1 / *cjphoX*	[36]
pAG101	pMA1 / *cjphoX:*C198A	This study
pAG102	pMA1 / *cjphoX:*C211A	This study
pAG103	pMA1 / *cjphoX:*C399A	This study
pAG104	pMA1 / *cjphoX:*C519A	This study
pAG105	pMA1 / *cjphoX:*C540A	This study

The *Escherichia coli* strains DH5α and TG1 served for the preparation of recombinant plasmids. The *E. coli* strain Rosetta (DE3) LacI^q^ was used to overproduce CjDsbA1 (pUWM837) and CjDsbA2 (pUWM1065). The *E. coli* strains JCB817 (*dsbA*) and JCB818 (*dsbA dsbB*) were employed for the CjDsbA1 (pUWM1246) complementation experiments.

*E. coli* strains were grown at 37°C on solid (1.5% agar), semisolid (0.35% agar) or liquid Luria-Bertani (LB) medium. *Campylobacter jejuni* strains were grown at 37°C on solid Blood Agar No. 2 (BA), semisolid (0.4% agar) Mueller-Hinton (MH), defined F12 medium [68] or in defined liquid medium [69] containing 0.08 mM phosphate [Pi], under microaerobic conditions generated by a gas pack system (Becton Dickinson) or by an anoxomat system (MART Microbiology). When appropriate, media were supplemented with antibiotics [ampicillin (100 µg ml^−1^), chloramphenicol (15 µg ml^−1^), kanamycin (30 or 40 µg ml^−1^)], Campylobacter Selective Supplement (Oxoid)], glucose (0.8–1% v/v), lactose (0.2% v/v), arabinose (0.2% v/v) and/or IPTG (3 mg ml^−1^) in DMF (dimethyl-formamide).

### Recombinant DNA techniques

Standard DNA manipulations were carried out as described previously [70] or according to the manufacturer's instructions (A&A Biotechnology). Polymerase chain reactions (PCR) were performed with *Taq* or HotStart High-Fidelity Polymerase (Qiagen) under standard conditions. Oligonucleotide primer synthesis (sequences given in Table 2) and DNA sequencing were performed by DNA Sequencing and Oligonucleotide Synthesis Laboratory at the Institute of Biochemistry and Biophysics, Polish Academy of Sciences, and by Genomed, Warsaw, Poland.

**Table 2:** Oligonucleotides used in the present study.

Name	Sequence	Restriction site
Cj17LSal	GCTGTCGAC **TGATAAGAAAGAATATTG**	**SalI**
Cj17RBgl	TTCAGATCT **CTAATGTGTTTAGCAGGC**	BglII
Cj872dw	*CTGCCCGGGATCGATGGATCCGTA* **TATCTCATAAACTTGCTGATG**	SmaI, ClaI, BamHI
Cj872up	*TACGGATCCATCGATCCCGGGCAG* **ATCTTTACCATTTTGCTC**	BamHI, ClaI, SmaI
Cj864LM	TGAGGATCC**ATGG** **AAAGCAAGCTAATG**	BamHI, NcoI
Cj864LS	GATCTAGA **CCTATTCTTGATTTTTAG**	XbaI
Cj864RM	GCAGAATTC **TCTTTAGTAATTTCAATC**	EcoRI
Cj864RS	TATGGATC**C** **TTTACCTTCACTTAATG**	BamHI
Cj864RX	CGCTCTAG**A** **AAGCAATGAATGTAAGTAA**	**XbaI**
Cj865RS	CAGGTCGACC**AATTATTTAAGACATCCTA**	**SalI**
Cjj882bis	**ATGGTTTATAGCGCAACAGC**	Ø
Cjj884_1	**TGCCTCAAGGTGCGCCTGAC**	Ø
C8J_0813_up	**TCTCTAACTCAATATGAA CC**	Ø
C8J_0814_dw2	**GTATCGTCTTATCAAAGCTG**	Ø
Cjj880Pag	GCGTCATGA **AAGGTAAAGAATATGTAATTC**	PagI
Cjj880Xh	GTGCTCGAG **TTGCTTGCTAAGTTCTTTAG**	XhoI
Cjj880B	ATCGGATCC**A** **CCTAGATTATTCTACTTTG**	BamHI
Cjj883Nc	GCCATGG **ATAATAGTTTTATTACTCTT**	NcoI
Cjj883Xh	GTGCTCGAG **TTTCATATTACTTAATTT**	XhoI
Cjj881_L	**TTATGGAACCTTGCGAACAATG**	Ø
Cjj881X	AGTTCTAG**A** **AAATGTGCTATACAAGTAAG**	XbaI
Cjj881_RT	ATAACAATCGCCAATGC	Ø
astA_Sal	TTAGTCGAC **ATGGTTATGTCTTAAGTG**	SalI
astA_Xba	GTTTCTAGA **ACTATCAATTCTCCAGCC**	XbaI
astA_Xba2	AGTTCTAGA **ATCATAATTCCACGATTG**	XbaI
astA_RT	TCTCTTCCTAAGATATCG	Ø
C1Afor	AATTTGTTCATGGAACGTTTGCAAATGCTGCAAATGGACAAACACC	AclI
C1Arev	GGTGTTTGTCCATTTGCAGCATTTGCAAACGTTCCATGAACAAATT	AclI
C2Afor	GGGAACTTATATCACAGCTGAAGAAAATTTTGATG	PvuII
C2Arev	CATCAAAATTTTCTTCAGCTGTGATATAAGTTCCC	PvuII
C3Afor	GTATTGTAGGTGCAACTCCCATGGATAGAGCTGAATGGATAGCAAGC	NcoI
C3Arev	GCTTGCTATCCATTCAGCTCTATCCATGGGAGTTGCACCTACAATAC	NcoI
C4Afor	CGAAAGTATGGGAAATAACGCCATGCTAGCAGCAAATCC	Ø
C4Arev	GGATTTGCTGCTAGCATGGCGTTATTTCCCATACTTTCG	Ø
C5Afor	GCTTTTTAACAGGGCCTATTGCAGCTGAATTAACAGGGATTGC	PvuII
C5Arev	GCAATCCCTGTTAATTCAGCTGCAATAGGCCCTGTTAAAAAGC	PvuII

**Bold letters** indicate *C. jejuni* nucleotide sequences; restriction recognition sites introduced for cloning purposes are underlined, complementary fragments of primers Cj872up and Cj872dw are marked with *italics*. Most primers were based on the *C. jejuni* 81116 nucleotide sequence, but for some experiments, previously designed primers based on the *C. jejuni* NCTC or 81–176 nucleotide sequences were used, or primers were designed to introduce point mutations. Their single pair mismatches with *C. jejuni* 81116 are double underlined.

### *C. jejuni* gene mutagenesis – allele exchange methodology

The *C. jejuni* 81116 mutants (*cjdsbA1*::*aphA-3*, *cjdsbA2::cat*, *cjdsbB::aphA-3* and *cjdsbI::cat*) were constructed for the purpose of this study, whereas a *C. jejuni* 81116 *cjphoX::cat* mutant was described previously [71]. For each of the obtained strains, we verified that the introduced mutation had no polar effect on the expression of adjacent genes.

To prepare a construct for *C. jejuni* 81116 *dsbA1* mutagenesis, both *cjdsbA1* gene arms were amplified from the *C. jejuni* genome, using two primer pairs (Cjj882bis - Cj872up and Cj872dw - Cjj884_1) designed to replace a 19 bp internal fragment from the *cjdsbA1* nucleotide coding sequence with restriction sites. Then, a third PCR reaction was performed using a mixture of two former PCR products (616 bp and 580 bp) as a template and the Cjj882bis - Cjj884_1 primer pair. The resulting PCR product (1214 bp) was cloned into pGEM-T Easy. Subsequently, a kanamycin resistance cassette (*aphA-3* gene excised from pBF14) was inserted between the *cjdsbA1* gene arms in the opposite transcriptional orientation, using BamHI endonuclease. The resulting plasmid was designated pUWM1306.

To prepare a construct for *C. jejuni* 81116 *dsbA2* mutagenesis, both *cjdsbA2* gene arms were amplified from the *C. jejuni* genome, using two primer pairs (Cj864LS - Cj864RS and Cj864LM - Cj864RM, respectively) designed to delete an internal 176 bp fragment from the *cjdsbA2* nucleotide coding sequence. PCR products (245 bp and 400 bp) were digested with XbaI - BamHI and BamHI - EcoRI enzyme pairs, respectively, and directionally cloned into pBluescript II SK. Subsequently, a chloramphenicol resistance cassette (*cat* gene excised from pRY109) was inserted between the *cjdsbA2* gene arms in the same transcriptional orientation, using BamHI endonuclease. The resulting plasmid was designated pUWM825.

Correct construction of respective pUWM1306 and pUWM825 was confirmed by restriction analysis and sequencing. Recombinant plasmids were electrotransformed into *C. jejuni* 81116 competent cells. The *C. jejuni cjdsbA1::aphA3* and *cjdsbA2::cat* mutants were verified by PCR analysis (using C8J_0813_up - C8J_0814_dw2 and Cjj880B – Cjj881X, respectively) and DNA sequencing.

The *C. jejuni* 81116 mutants in the *dsbB* and *dsbI* genes were constructed using plasmids pUWM607 (*cjdsbB::aphA3*) and pUWM713 (*cjdsbI::cat*), respectively, as previously described for *dsbB* / *dsbI* mutagenesis of the *C. jejuni* 81–176 strain [25], [28]. Recombinant plasmids were electrotransformed into *C. jejuni* 81116 competent cells. The *C. jejuni cjdsbB::aphA3* and *cjdsbI::cat* mutants were verified by PCR analysis (using, respectively, Cj864RX - Cj865RS and Cj17LSal - Cj17RBgl primer pairs) and DNA sequencing.

### Construction of the *cjdsbA1^+^* plasmid for *C. jejuni dsbA1^-^* complementation experiments

To analyze the complementation of the *cjdsbA1* mutation in *C. jejuni* 81116, a recombinant plasmid was constructed based on the *E. coli*/*C. jejuni* shuttle plasmid, pRY111. The nucleotide sequence containing the *cjdsbA1* gene, with its own promoter, was amplified from *C. jejuni* 81116 genomic DNA using a pair of primers: C8J_813_up and C8J_814_dw. The PCR product (1066 bp) was first cloned into the pGEM-T Easy vector, resulting in construction of plasmid pUWM1213. Thereafter, the insert was cut out with EcoRI enzyme and cloned into pRY111. The resulting plasmid was designated pUWM1214 and its correct construction was confirmed by restriction analysis and sequencing. Subsequently, pUWM1214 was introduced into *C. jejuni* 81116 lacking *cjdsbA1,* and the resulting strain was used for the DsbA1 complementation tests.

### Site-directed mutagenesis of the *cjphoX*

To identify the cysteine residues of CjPhoX linked by disulfide bonds, a set of recombinant plasmids was constructed from pMA1 carrying the *cjphoX* gene with its own promoter. The Cys-to-Ala point mutations were generated using the Quick Change Site-Directed Mutagenesis Kit (Qiagen) according to the manufacturer's instructions, starting with 100 ng of pMA1 template and 125 ng of each primer (primer pairs: C1Afor – C1Arev, C2Afor – C2Arev, C3Afor – C3Arev, C4Afor – C4Arev, C5Afor – C5Arev). The resulting plasmids were designated pAG101-pAG105, respectively and their correct construction was confirmed by sequencing. Subsequently the plasmids pAG101-pAG105 were introduced into *C. jejuni* 81116 lacking *cjphoX,* and the resulting strains were used for the PhoX activity assays.

### Construction of the *cjdsbA1^+^* plasmid for *E. coli dsbA* ^-^ and *E. coli dsbA^-^ dsbB^-^* complementation experiments

Previous experiments ([25] and unpublished data) demonstrated that native promoter region and the signal sequence (SS) of CjDsbA1 are not recognized and processed in *E. coli* cells. Therefore, the CjDsbA1 gene, excised from pUWM837 (*ss*'*pelB-cjdsbA1-his6*; see below) with NdeI/Klenow fragment and subsequently with XhoI endonuclease, was cloned into a pMPM-A6 [72] vector cut with EcoRI/Klenow fragment and XhoI endonuclease, which placed it under the arabinose promoter. Correct construction of the obtained pUWM1246 plasmid was confirmed by restriction analysis and DNA sequencing. The recombinant plasmid was transformed into *E. coli* JCB817 (*dsbA::aphA3*) and JCB818 (*dsbA::kan1 dsbB::kan*) *E. coli* strains. Production of the recombinant CjDsbA1 protein was confirmed by Western-blot analysis of cell extracts with anti-DsbA1 and anti-6xHis sera.

### RT-PCR

To confirm the specific character of *cjdsbA2* and *cjdsbB* mutations, we analyzed the transcription level of their downstream genes, compared to the wild type strain, by means of RT-PCR (reverse transcription - PCR) carried out as previously described [25]. Total RNAs were extracted from the *C. jejuni* 81116 strains using the standard TRIzol procedure (Invitrogen). After DNase I treatment, RNA was reverse-transcribed using Omniscript Reverse Transcriptase (Qiagen) and a primer specific for *cjdsbB* or *cjastA*, i.e., Cjj881_RT or astA_RT, respectively. PCR reactions without reverse transcriptase were used to confirm that the RNA was free of DNA contamination. PCR reactions performed on cDNA were carried out with a pair of primers, Cjj881L - Cjj881R (complementary to the *cjdsbB*; expected product 465 bp) or astA_Sal - astA_Xba2 (complementary to the *cjastA* gene; expected product 516 bp).

### Protein analysis

Preparation of *C. jejuni* and *E. coli* protein extracts, SDS-PAGE (sodium dodecyl sulfate polyacrylamide gel electrophoresis) and blotting procedures were performed by standard techniques [70].

### Overexpression and purification of CjDsbA1, CjDsbA2 and EcDsbA

To obtain recombinant CjDsbA1/CjDsbA2 proteins, two constructs were prepared, each carrying translational fusions in which the original *C. jejuni* signal sequences (i.e., 31 and 26 N-terminal amino acids, respectively (SignalP server)) were replaced by an N-terminal *E. coli* PelB-signal sequence (SS PelB) [73]. *C. jejuni* DNA fragments of 575 bp and 578 bp were PCR-amplified using the primer pairs Cjj883Nc - Cjj883Xh and Cjj880Pag - Cjj880Xh, respectively, and cloned into the pET22b expression vector using NcoI/PagI and XhoI restriction enzymes, to generate the pUWM837 and pUWM1065 recombinant vectors, respectively. Overproduction of periplasmic soluble protein was obtained under autoinducing conditions, as described by Studier *et al.* [74]. Recombinant proteins (rCjDsbA1 and rCjDsbA2) were purified by affinity chromatography. EcDsbA was overproduced and purified from recombinant plasmid pDEST14-DsbA, kindly provided by J.F. Collet, as described previously [27]. CjDsbA1, CjDsbA2 and EcDsbA were used in the insulin reduction assay, and CjDsbA1 was also used for rabbit immunization.

### Phenotype assays

To determine the role of the CjDsbAs in cell biology and their relative contributions to the oxidative folding of *C. jejuni* proteins, we studied the effects of *cjdsbA1* and *cjdsbA2* mutations on motility and autoagglutination (AAG), mechanisms that are necessary for bacteria to colonize and spread within the host organism [75]. We subsequently examined the impact of each CjDsbA on the enzymatic activity of two Dsb substrate proteins, arylsulfotransferase CjAstA and alkaline phosphatase CjPhoX [22], [30].

### Motility assay

Motility of *Campylobacter* strains on soft agar was assessed as previously described [76]. Briefly, *C. jejuni* were grown for 16 hours on BA plates, harvested and diluted in LB to an optical density of 0.6 at 600 nm (OD_600_). Aliquots of 2 µl were spotted onto the surface of semisolid MH medium supplemented with 0.4% agar. Alternatively, *C. jejuni* were grown 16 hours on F12 plates, harvested and diluted in PBS and aliquots spotted on semisolid 0.4% F12-agar medium. Motility plates were incubated for 48 h at 37°C under microaerobic conditions. The low density of the agar allows the bacteria to move within the agar, forming a halo of growth around the point of inoculation.

Motility of *E. coli*-complemented strains was assessed as described previously [27]. Briefly, *E. coli* strains were freshly grown until logarithmic phase (an optical density of 0.6 at 600 nm (OD_600_) in LB medium supplemented with arabinose (0.2% v/v). Aliquots of 2 µl were spotted onto the surface of semisolid LB medium supplemented with arabinose and 0.35% agar. Motility plates were incubated for 24–48 h at 37°C.

### Autoagglutination (AAG) assay

Autoagglutination was measured as described previously [76]. *C. jejuni* was grown for 16 hours on BA plates, harvested and diluted in LB to an optical density of 0.6 at 600 nm (OD_600_). The prepared bacterial suspension was kept undisturbed at room temperature overnight. Strains capable of AAG fell to the bottom of the tube, leaving a clear supernatant. The degree of AAG was quantitated by removal of the top 1 ml of the suspension and measurement of the OD_600_.

## Biochemical assays

### Enzymatic assays

Qualitative assays for AstA (arylsulfatase) activity were carried out as described previously [77]. Briefly, *C. jejuni* were grown for 16 hours on MH plates supplemented with XS (5-bromo-4-chloro-3-indolylsulfate, 100 µg ml^−1^), a substrate for arylsulfatase, and bacterial colonies were observed for blue color acquisition, which indicates AstA activity. Quantitative assays for AstA activity in *C. jejuni* strains were performed in triplicate, using a modified method described by Hendrixson and DiRita [78].

The PhoX (alkaline phosphatase) activity in *C. jejuni* strains was determined on defined medium [69] as described by van Mourik *et al.* [36], and quantified by monitoring the release of p-nitrophenol from p-nitrophenyl phosphate (PNPP) (Sigma).

### Determination of the CjDsbA1 redox state

The redox state of CjDsbA1 was visualized by alkylating the free cysteine residues using 4-acetamido-4′-maleimidylstilbene-2,2′-disulfonic acid (AMS), resulting in a molecular mass increase of 0.5 kDa. Briefly, bacteria harvested from an overnight microaerobic BA plate culture were washed twice and resuspended in PBS to obtain OD_600_ 0.1 for a 10^−2^ dilution. Proteins were precipitated with TCA (final concentration of 5%), washed twice with cold acetone (200 µl) and dried. Subsequently, the protein pellets were resuspended in 100 µl of freshly prepared AMS buffer (50 mM Tris-HCl, 10 mM AMS, 1% SDS, 1 mM EDTA; pH 8.0) and stored for 20 min at room temperature. Proteins were precipitated for 30 min at 4°C by the addition of 1 ml of cold acetone, centrifuged (20 min, 14000 rpm), dried and resuspended in non-reducing loading buffer (125 mM Tris-HCl, 4% SDS, 20% glycerol; pH 6.8). Samples obtained from cells pre-treated 30 min at 30°C with 100 µl of 1 M DTT (dithiothreitol) before TCA precipitation served as the control for the reduced sulfhydryl groups. Samples were stored at −20°C until electrophoretic analysis.

### Insulin reduction assay

The ability of CjDsbA1, CjDsbA2 and EcDsbA to catalyze the reduction of insulin in the presence of DTT was determined as previously described [30], [79] using human insulin solution (Sigma). Reactions (triplicate) were carried out in 200 µl of 100 mM sodium phosphate buffer, pH 7.0, 131 µM insulin, 0.33 mM dithiothreitol (DTT), and 10 µM CjDsbA1, CjDsbA2 or EcDsbA solution; incubated at room temperature in a 96-well plate. Reactions started by adding DTT to the final concentration of 0.33 mM. The changes in the absorbance at 650 nm as a function of time were measured in Sunrise (Tecan) plate reader.

## Supporting Information

Figure S1 **Cluster map of DsbA family.** Dots correspond to protein sequences and are arranged on the map according to pairwise similarities measured with BLAST. Clusters DsbA and DsbA1–2 used in further sequence analyses (Figure 1 and Figures S3 and S4) are indicated with labels.
(TIF)

Figure S2 **Cluster map of DsbB family.** Dots correspond to protein sequences and are arranged on the map according to pairwise similarities measured with BLAST. Clusters DsbB and DsbB2 and DsbI used in further sequence analyses (Figure 1 and Figures S3 and S4) are indicated with labels.
(TIF)

Figure S3 **Phylogenetic tree of the classical DsbA and DsbA1–2 clusters.** Two main clades were defined: first comprises sequences from Gammaproteobacteria, whereas the second – sequences mostly from Epsilonproteobacteria. Within the second clade a group of Gammaproteobacterial sequences were identified, indicating a horizontal gene transfer from Epsilonproteobacteria to Gammaproteobacteria. The tree supports the notion that CjDsbA1 and CjDsbA2 are closely related paralogs. Localization of the proteins discussed in the text is indicated with red labels. Numbers at the nodes indicate the Fasttree2 support values.
(TIF)

Figure S4 **Phylogenetic tree of the classical DsbB and DsbB2 clusters.** Two main clades were defined: first comprises sequences from Gammaproteobacteria, whereas the second – sequences mostly from Epsilonproteobacteria. Within the second clade a group of Gammaproteobacterial sequences were identified, indicating a horizontal gene transfer from Epsilonproteobacteria to Gammaproteobacteria. Localization of the proteins discussed in the text is indicated with red labels. Numbers at the nodes indicate the Fasttree2 support values.
(TIF)

Figure S5 **Phylogenetic tree of the arylsulfotransferase AstA. Proteins from distantly related Gammaproteobacteria and Epsilonproteobacteria are localized together on the tree suggesting a horizontal gene transfer event. AstA tree, in contrast to DsbA and DsbB trees, does not clearly identify the direction of the transfer. Numbers at the nodes indicate the Fasttree2 support values.**
(TIF)

Figure S6 **Motility and autoagglutination of** ***C. jejuni*** **81116 strains: wild type (WT),** ***cjdsbA1^-^*** **and** ***cjdsbB^-^*** **mutants grown in defined medium F12.** Bacterial motility (A) was monitored after 24 hours of incubation on 0.4% F12-agar plates. The *cjdsbA1^-^* strain is non-motile, contrary to the wild type (WT) and *cjdsbB^-^* strains. Bacterial autoagglutination was monitored as a decrement of turbidity (B) or optical density (C) of bacterial suspension in PBS at room temperature after harvesting cells from F12 plates. The *cjdsbA1^-^* strain does not autoagglutinate, contrary to the wild type (WT) and *cjdsbB^-^* strains. The figure presents a representative result.
(TIF)

Figure S7 **Alkaline phosphatase PhoX activity in** ***C. jejuni*** **81116 strains: wild type,** ***cjphoX*** **mutant and** ***cjphoX*** **mutant complemented** ***in trans*** **by wild type (WT) and point mutated plasmid version of** ***cjphoX*** **: C198A (C1A), C211A (C2A), C399A (C3A), C519A (C4A) and C540A (C5A).** The diagrams illustrate mean values and standard deviations of PhoX activity derived from three experiments; for each experiment the PhoX activity were carried out in duplicate. Statistical significance was calculated using Student *t* test for comparison of independent groups (GraphPad Prism) with reference to the PhoX activity in the *cjphoX^-^* mutant strain complemented with wild type *phoX* (*phoX WT*). P values of P<0.05 were considered statistically significant (*).
(TIF)

Figure S8 **Model representation of Dsb proteins functioning in oxidative protein folding in** ***C. jejuni*** **cells.**
(TIF)
