# Supplementary figures and images for: Functional and Bioinformatics Analysis of Two Campylobacter jejuni Homologs of the Thiol-Disulfide Oxidoreductase, DsbA
Source: PLoS One. 2014 Sep 2;9(9):e106247. doi: 10.1371/journal.pone.0106247 (PMC4152235; doi:10.1371/journal.pone.0106247)

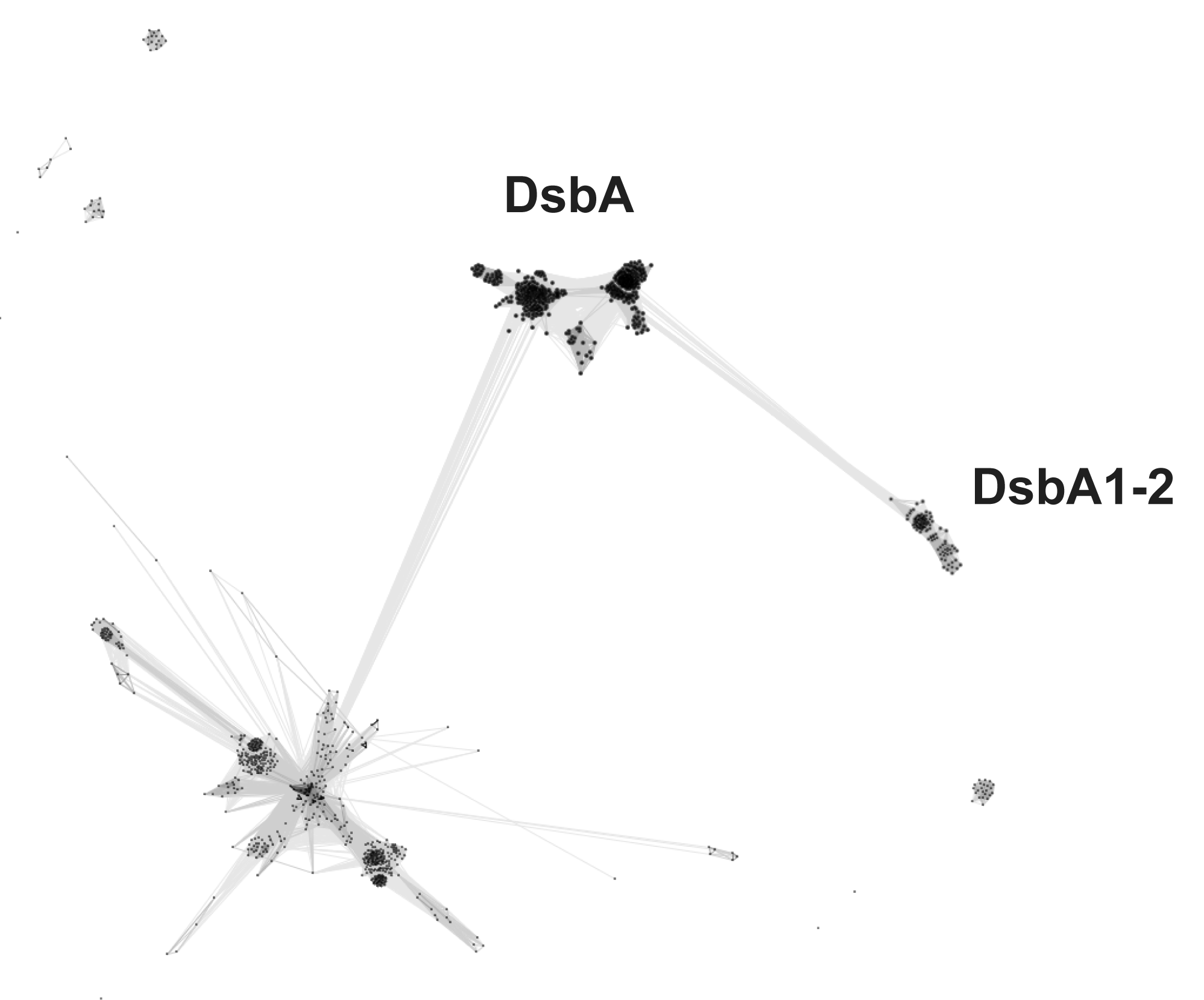

Supplement: Figure S1 — Cluster map of DsbA family. Dots correspond to protein sequences and are arranged on the map according to pairwise similarities measured with BLAST. Clusters DsbA and DsbA1–2 used in further sequence analyses (Figure 1 and Figures S3 and S4) are indicated with labels. (TIF) [file pone.0106247.s001.tif]

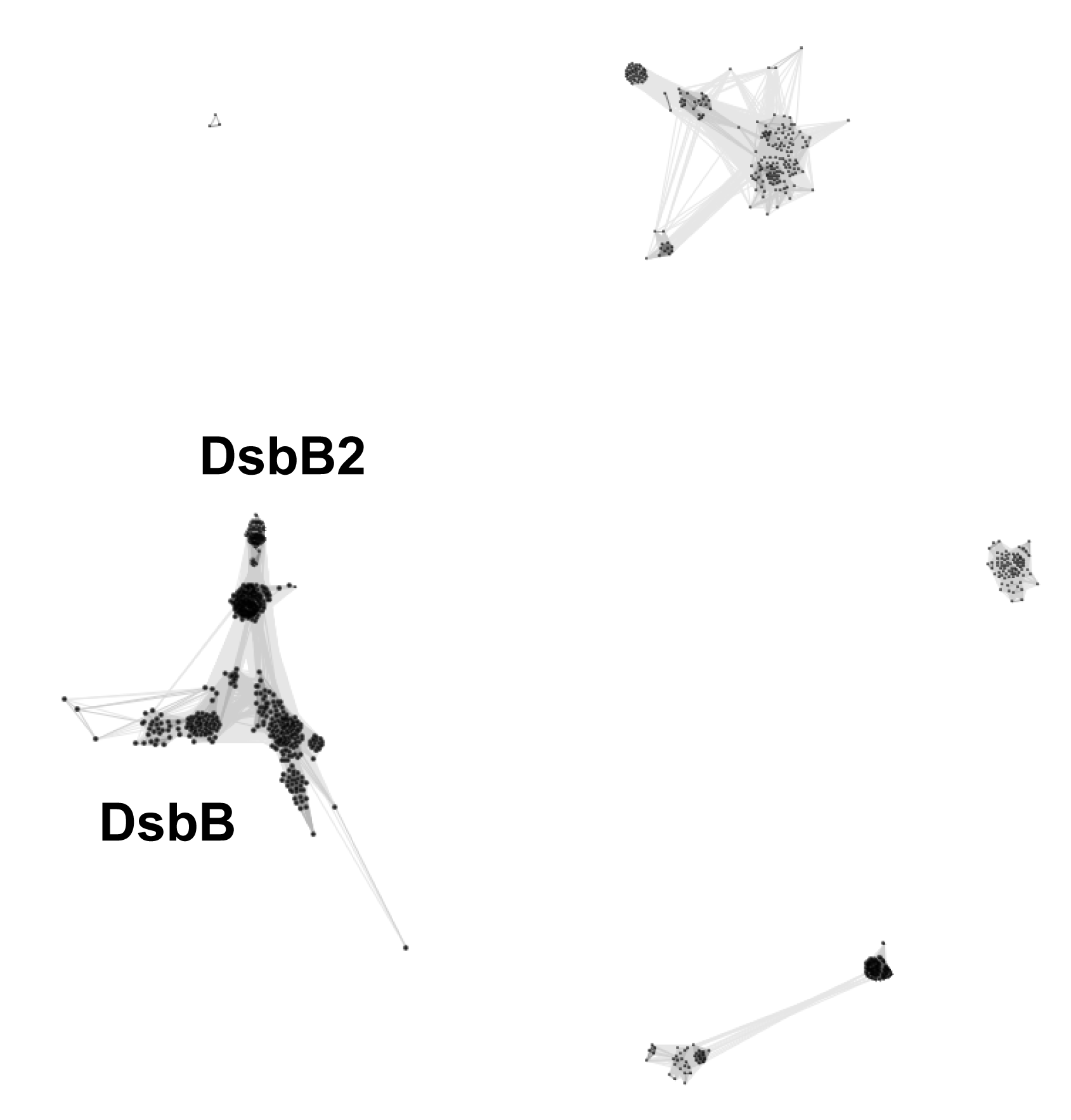

Supplement: Figure S2 — Cluster map of DsbB family. Dots correspond to protein sequences and are arranged on the map according to pairwise similarities measured with BLAST. Clusters DsbB and DsbB2 and DsbI used in further sequence analyses (Figure 1 and Figures S3 and S4) are indicated with labels. (TIF) [file pone.0106247.s002.tif]

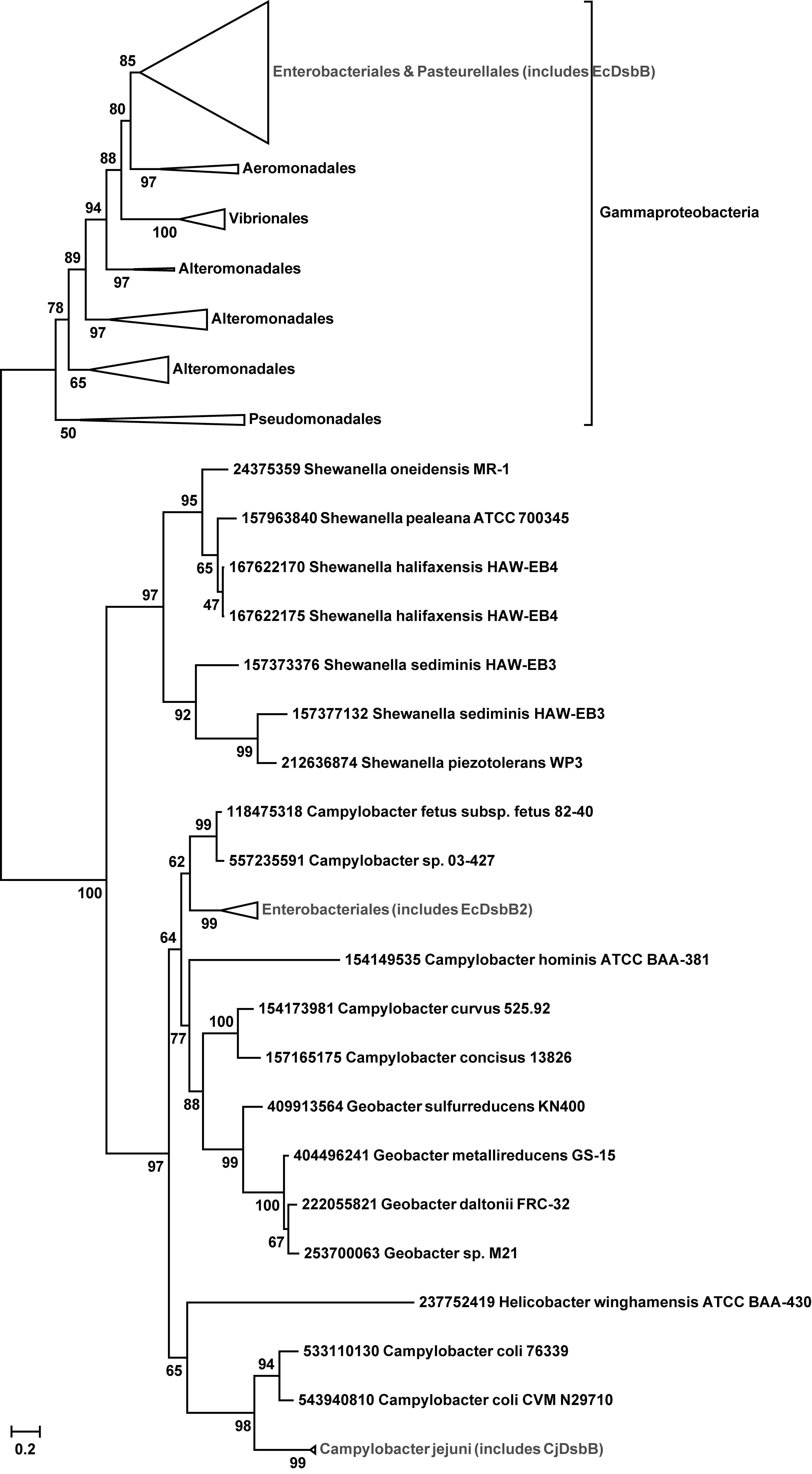

Supplement: Figure S4 — Phylogenetic tree of the classical DsbB and DsbB2 clusters. Two main clades were defined: first comprises sequences from Gammaproteobacteria, whereas the second – sequences mostly from Epsilonproteobacteria. Within the second clade a group of Gammaproteobacterial sequences were identified, indicating a horizontal gene transfer from Epsilonproteobacteria to Gammaproteobacteria. Localization of the proteins discussed in the text is indicated with red labels. Numbers at the nodes indicate the Fasttree2 support values. (TIF) [file pone.0106247.s004.tif]

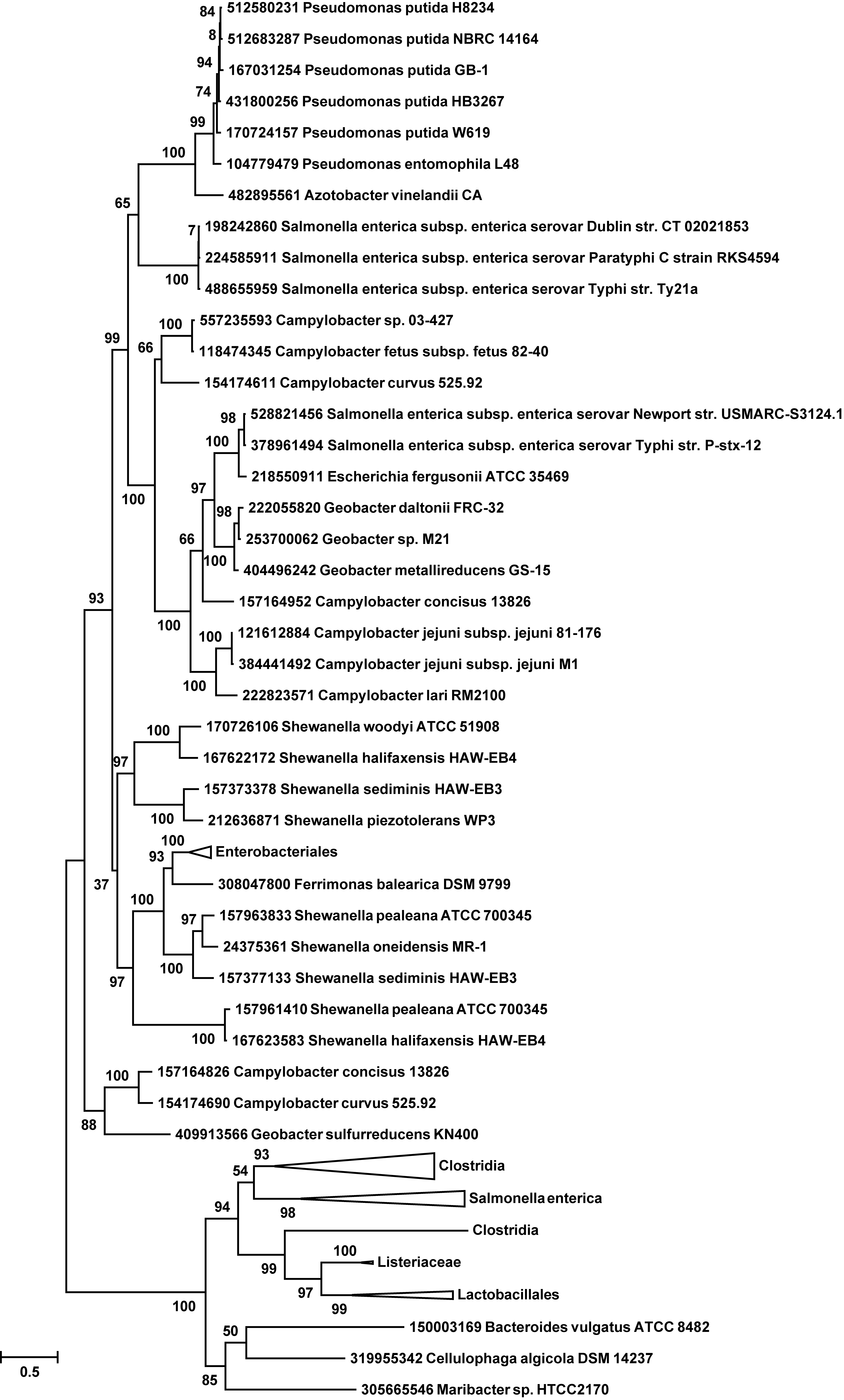

Supplement: Figure S5 — Phylogenetic tree of the arylsulfotransferase AstA. Proteins from distantly related Gammaproteobacteria and Epsilonproteobacteria are localized together on the tree suggesting a horizontal gene transfer event. AstA tree, in contrast to DsbA and DsbB trees, does not clearly identify the direction of the transfer. Numbers at the nodes indicate the Fasttree2 support values. (TIF) [file pone.0106247.s005.tif]

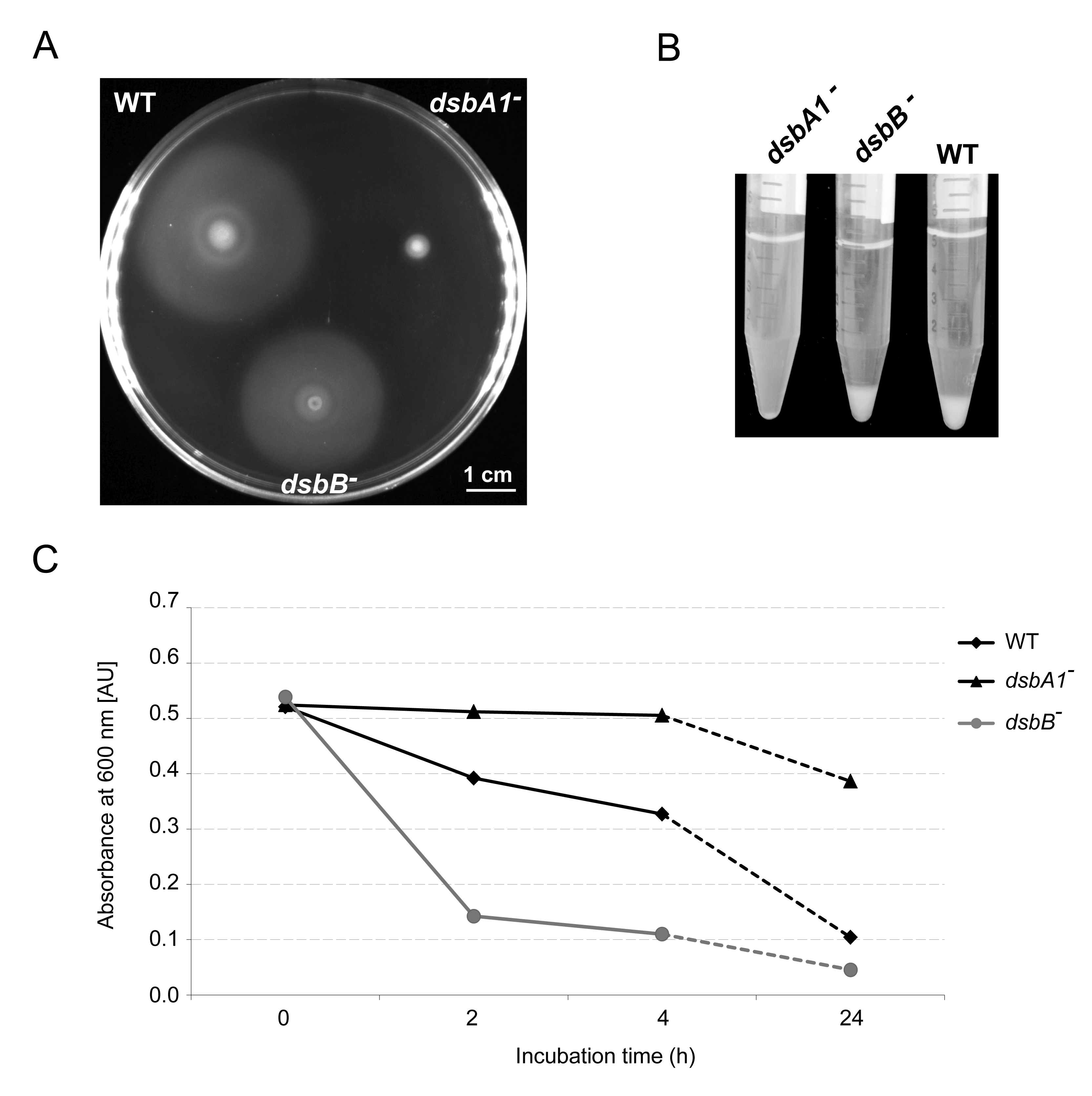

Supplement: Figure S6 — Motility and autoagglutination of C. jejuni 81116 strains: wild type (WT), cjdsbA1- and cjdsbB- mutants grown in defined medium F12. Bacterial motility (A) was monitored after 24 hours of incubation on 0.4% F12-agar plates. The cjdsbA1- strain is non-motile, contrary to the wild type (WT) and cjdsbB- strains. Bacterial autoagglutination was monitored as a decrement of turbidity (B) or optical density (C) of bacterial suspension in PBS at room temperature after harvesting cells from F12 plates. The cjdsbA1- strain does not autoagglutinate, contrary to the wild type (WT) and cjdsbB- strains. The figure presents a representative result. (TIF) [file pone.0106247.s006.tif]

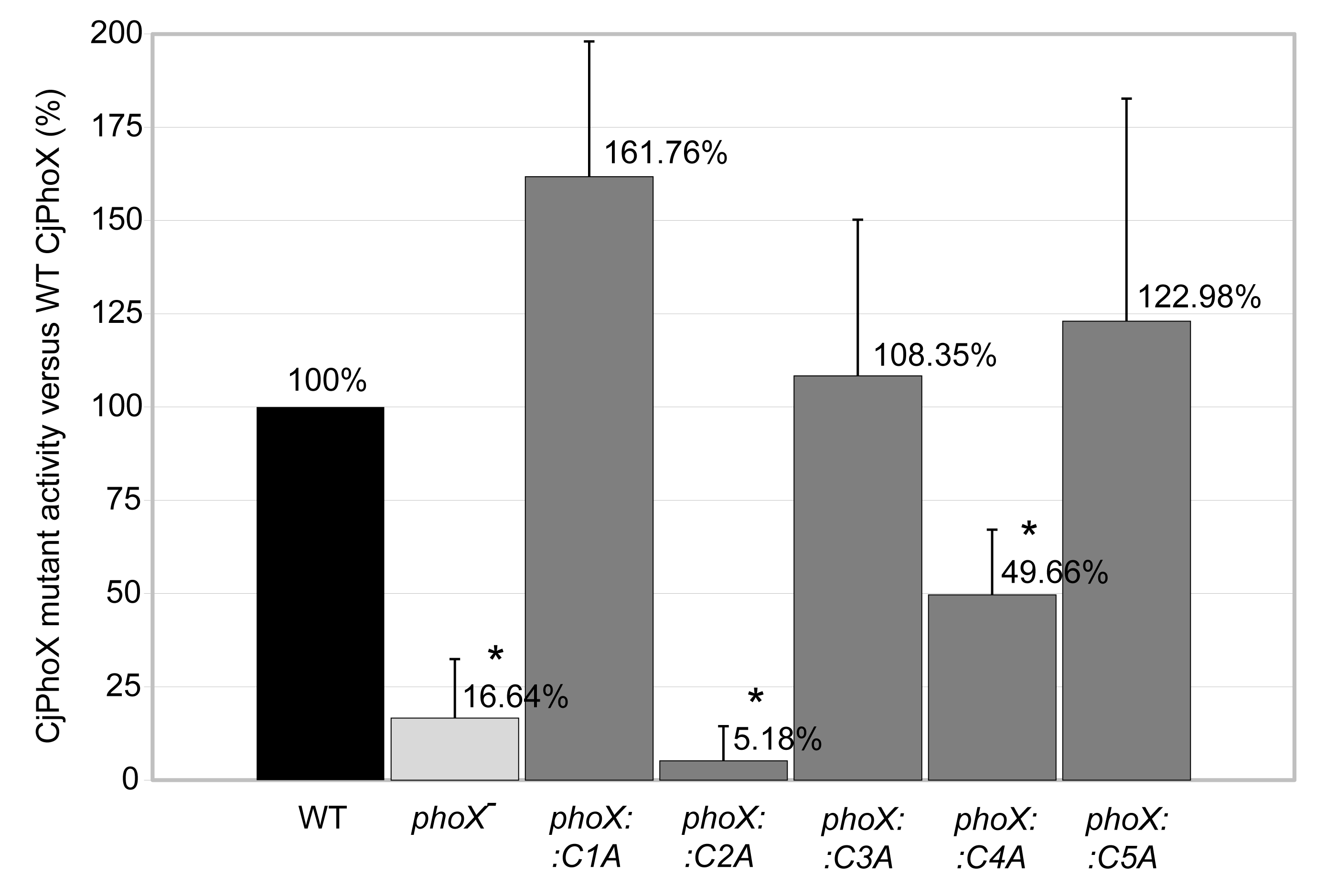

Supplement: Figure S7 — Alkaline phosphatase PhoX activity in C. jejuni 81116 strains: wild type, cjphoX mutant and cjphoX mutant complemented in trans by wild type (WT) and point mutated plasmid version of cjphoX : C198A (C1A), C211A (C2A), C399A (C3A), C519A (C4A) and C540A (C5A). The diagrams illustrate mean values and standard deviations of PhoX activity derived from three experiments; for each experiment the PhoX activity were carried out in duplicate. Statistical significance was calculated using Student t test for comparison of independent groups (GraphPad Prism) with reference to the PhoX activity in the cjphoX- mutant strain complemented with wild type phoX (phoX WT). P values of P<0.05 were considered statistically significant (*). (TIF) [file pone.0106247.s007.tif]

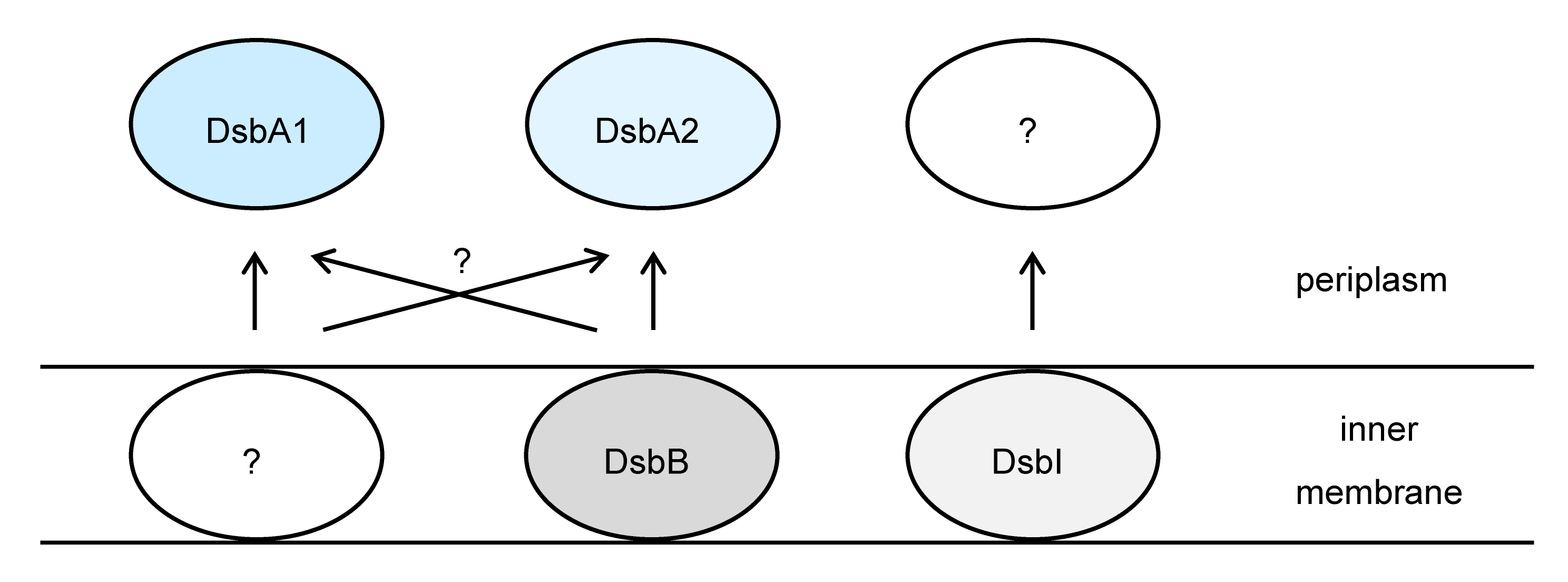

Supplement: Figure S8 — Model representation of Dsb proteins functioning in oxidative protein folding in C. jejuni cells. (TIF) [file pone.0106247.s008.tif]
